# The regulation of Hypoxia-Inducible Factor-1 (HIF-1alpha) expression by Protein Disulfide Isomerase (PDI)

**DOI:** 10.1371/journal.pone.0246531

**Published:** 2021-02-04

**Authors:** Yukino Kobayashi, Ami Oguro, Yuta Hirata, Susumu Imaoka

**Affiliations:** 1 Department of Biomedical Chemistry, School of Science and Technology, Kwansei Gakuin University, Gakuen, Sanda, Japan; 2 Program of Biomedical Science, Graduate School of Integrated Sciences for Life, Hiroshima University, Higashihiroshima, Japan; Universidade de Sao Paulo Instituto de Biociencias, BRAZIL

## Abstract

Hypoxia-inducible factor-1alpha (HIF-1alpha), a transcription factor, plays a critical role in adaption to hypoxia, which is a major feature of diseases, including cancer. Protein disulfide isomerase (PDI) is up-regulated in numerous cancers and leads to cancer progression. PDI, a member of the TRX superfamily, regulates the transcriptional activities of several transcription factors. To investigate the mechanisms by which PDI affects the function of HIF-1alpha, the overexpression or knockdown of PDI was performed. The overexpression of PDI decreased HIF-1alpha expression in the human hepatocarcinoma cell line, Hep3B, whereas the knockdown of endogenous PDI increased its expression. NH_4_Cl inhibited the decrease in HIF-1alpha expression by PDI overexpression, suggesting that HIF-1alpha was degraded by the lysosomal pathway. HIF-1alpha is transferred to lysosomal membranes by heat shock cognate 70 kDa protein (HSC70). The knockdown of HSC70 abolished the decrease, and PDI facilitated the interaction between HIF-1alpha and HSC70. HIF-1alpha directly interacted with PDI. PDI exists not only in the endoplasmic reticulum (ER), but also in the cytosol. Hypoxia increased cytosolic PDI. We also investigated changes in the redox state of HIF-1alpha using PEG-maleimide, which binds to thiols synthesized from disulfide bonds by reduction. An up-shift in the HIF-1alpha band by the overexpression of PDI was detected, suggesting that PDI formed disulfide bond in HIF-1alpha. HIF-1alpha oxidized by PDI was not degraded in HSC70-knockdown cells, indicating that the formation of disulfide bond in HIF-1alpha was important for decreases in HIF-1alpha expression. To the best of our knowledge, this is the first study to show the regulation of the expression and redox state of HIF-1alpha by PDI. We also demonstrated that PDI formed disulfide bonds in HIF-1alpha 1–245 aa and decreased its expression. In conclusion, the present results showed that PDI is a novel factor regulating HIF-1alpha through lysosome-dependent degradation by changes in its redox state.

## Introduction

Hypoxia-inducible factor-1alpha (HIF-1alpha) is a key transcription factor in the hypoxia response, which is common in ischemic diseases, including cancer. HIF-1alpha is up-regulated in various cancers and induces angiogenesis, alterations in metabolism, metastasis, migration, and invasion in cancer [1, 2]. HIF-1alpha has also been shown to induce various angiogenetic factors, including vascular endothelial growth factor (VEGF) and angiopoietins [3, 4]. Angiogenesis enables cancer growth by supplying oxygen and nutrients [3, 4]. HIF-1alpha increases glucose uptake by inducing glucose transporter 1 (Glut1) and Glut 3 [3, 4]. Furthermore, HIF-1alpha induces pyruvate dehydrogenase kinase (PDK) [3, 4]. PDK inhibits the conversion of pyruvate to acetyl-CoA by the phosphorylation and inactivation of pyruvate dehydrogenase (PDH) [3, 4]. Therefore, HIF-1alpha enhances the shift from oxidative phosphorylation to anaerobic glycolysis and suppresses oxygen consumption. Matrix metalloproteinase-2 (MMP-2) and MMP-9, which degrade components of the extracellular matrix (ECM), are induced in a HIF-1alpha-dependent manner [5]. The degradation of ECM by MMPs enables cancer invasion and metastasis [5].

In addition to HIF-1alpha, protein disulfide isomerase (PDI) levels are elevated in various types of cancers [6, 7]. PDI is involved in the metastasis, migration, and survival of cancer [6, 7]. It also catalyzes the oxidation, reduction, and isomerization of nascent and misfolded proteins [8]. Increased levels of folding enzymes, such as PDI, are needed in cancer cells with high proliferation rates [9]. PDI inhibitors accumulate misfolded proteins, which induces apoptosis in cancer cells via ER stress [10]. Besides protein folding, PDI regulates protein functions via dithiol-disulfide exchange. It activates and enhances the secretion of MMPs by dithiol-disulfide exchange, thereby enhancing cancer cell invasion and metastasis [6, 11]. PDI also changes the conformation of beta3 integrin via dithiol-disulfide exchange and enhances adhesion [12]; therefore, PDI may enhance cancer cell migration by activating beta3 integrin [13, 14]. Bacitracin, an inhibitor of PDI activity, and beta3 integrin antibodies have been shown to inhibit cancer cell migration [13]. Recent studies identified PDI as a useful target for cancer therapy [15]; however, the mechanisms underlying the progression of cancer by PDI are highly complex and have not yet been elucidated in detail.

HIF-1alpha is modified by prolyl hydroxylase domain-containing protein (PHD) in an oxygen-dependent manner. Under normoxic conditions, HIF-1alpha is hydroxylated by PHD, and von Hippel-Lindau tumor suppressor protein (pVHL) induces the ubiquitination and proteasomal degradation of HIF-1alpha [1]. Under hypoxic conditions, PHD activity is suppressed by decreases in molecular oxygen, and HIF-1alpha is stabilized and binds to hypoxia response element (HRE), which induces the expression of HIF-1alpha target genes [1]. In addition to PHD, HIF-1alpha expression is regulated by a number of post-translational modifications, including phosphorylation, methylation, acetylation, S-nitrosylation, and SUMOylation, via its amino acid residues [16–28]. HIF-1alpha is also regulated by cellular redox factors. Thioredoxin (TRX) increases HIF-1alpha expression, whereas the catalytically inactive mutant of TRX exerts the opposite effect under normoxic and hypoxic conditions [29]. Buthionine sulphoximine (BSO) increases the expression of reduced glutathione (GSH), leading to the destabilization of HIF-1alpha in HepG2 cells [30]. Previous studies that investigated the effects of hydrogen peroxide (H_2_O_2_) on HIF-1apha expression reported contradictory findings, with increases [31] and decreases [32] both being reported. The molecular mechanisms underlying the redox regulation of HIF-1alpha currently remain unclear.

Since PDI has an endoplasmic reticulum (ER) retrieval sequence at its C terminus, it mainly localizes in the ER [8]. PDI has also been detected in the cytosol and nucleus and on the cell surface [33]. Several transcription factors are regulated by PDI redox activity. For example, PDI has been shown to promote the DNA binding of nuclear factor-kappa B (NF-kappa B) and activator protein-1 (AP-1), and its activity is enhanced by GSH, suggesting that the reduction of these transcription factors by PDI is required for DNA binding [34]. PDI also prevents the oxidation of estrogen receptor alpha, which stabilizes the zinc finger in the DNA-binding domain of estrogen receptor alpha and enhances DNA binding [35]. We previously reported that the overexpression of PDI inhibites the transcriptional activity of thyroid hormone receptor (TR), which is a substrate of redox factor-1 (Ref-1), by the oxidation of cysteine residues in Ref-1 in the rat pituitary tumor cell line, GH3 [36]. Ref-1 has been shown to activate HIF-1alpha transcriptional activity by enhancing the interaction between HIF-1alpha and creb-binding protein (CBP)/p300 [37].

HIF-1alpha and PDI are both up-regulated in various cancer cells and play roles in cancer progression. However, it currently remains unclear whether they cooperate or compete in cancer cells. Therefore, the aim of the present study was to elucidate the role of PDI in HIF-1alpha regulation in cancer cells. We investigated the effects of increased PDI expression levels on HIF-1alpha in the human hepatocarcinoma cell line, Hep3B. We found that the overexpression of PDI decreased HIF-1alpha expression in a lysosome-dependent manner. The degradation of HIF-1alpha was attributed to an increase in its oxidation state by cytosolic PDI.

## Experimental procedures

### Materials

DTT, dipyridyl, NH_4_Cl, an anti-DYKDDDDK (FLAG) tag antibody, an anti-Myc tag antibody, leupeptin, and Dulbecco’s modified Eagle’s medium (DMEM) were purchased from Wako Pure Chemical Industries, Ltd. (Osaka, Japan). Diamide, *N*-ethylmaleimide (NEM), methoxypolyethylene glycol (PEG) maleimide, fetal bovine serum (FBS), penicillin-streptomycin solution, and S-methyl methanethiosulfonate (MMTS) were obtained from Sigma-Aldrich (St. Louis, MO). (*N*-[6-(biotinamido)hexyl]-3’-(2’-pyridyldithio) propionamide (Biotin-HPDP) and streptavidin-agarose were purchased from Pierce Biotechnology (Rockford IL). An anti-HSC70 antibody was purchased from Novus Biologicals (Littleton, CO). An anti-BiP antibody was purchased from GeneTex, Inc. (Irvine, CA). Dimethyloxaloylglycine (DMOG), diamide, and a horseradish peroxidase (HRP) Maleimide Conjugate were purchased from Tokyo Chemical Industry Co., Ltd. (Tokyo, Japan). KOD plus neo DNA polymerase was purchased from TOYOBO (Tokyo, Japan). Revert AidTM M-MuLV Reverse transcriptase was purchased from MBI Fermentas (Vilnius, Lithuania).

### Cell culture and transfection

The human hepatocarcinoma cell line, Hep3B was obtained from the Cell Resource Center for Biomedical Research at the Institute of Development, Aging, and Cancer of Tohoku University (Miyagi, Japan). Hep3B cells, the human embryonic kidney cell line, HEK293T, and the cell line derived human cervix carcinoma, HeLa, were cultured in DMEM containing 10% FBS, penicillin (100 units/mL), and streptomycin (100 μg/mL). HEK293 cells were cultured in DMEM/Ham’s F-12 containing 10% FBS, penicillin (100 units/mL), and streptomycin (100 μg/mL). Cells were maintained at 37°C in 5% CO_2_ and 95% air (normoxia). In the hypoxic stimulation, cells were cultured in a multi-gas incubator (APM-30D, Astec, Shizuoka, Japan) set to 1% O_2_ and 5% CO_2_ (hypoxia). Regarding the overexpression of the wild type (WT) and cysteine mutant of PDI, HSC70, the WT and lysine mutant of HIF-1alpha, the WT and cysteine mutant of Ero-1, and HIF-1alpha (1–245 aa), cells were transfected with the plasmids with calcium phosphate.

### Preparation of constructs

Human PDI cDNA in a pcDNA3.1 (+) vector (Invitrogen, Carlsbad, CA) was constructed as described previously [38]. Human HSC70 cDNA in a pcDNA3.1 (+) vector was also constructed as described previously [39]. Human PDI C53, 397S, human HIF-1alpha WT, HIF-1alpha K719T, human Ero-1 WT, Ero-1 C104, 131S, Ero-1 C394, 397S, Ero-1 (24–469 aa), HIF-1alpha (1–245 aa), HIF-1alpha (245–826 aa), HIF-1alpha C90S (1–245 aa), HIF-1alpha C210S (1–245 aa), and HIF-1alpha C219S (1–245 aa) cDNA were amplified by PCR. The cDNA obtained from the total RNA of Hep3B cells was used as a template. All primers were indicated in Table 1. PCR for PDI C53, 397S, HIF-1alpha WT, and HIF-1alpha K719T cDNA was performed using KOD plus neo DNA polymerase with denaturation at 94°C for 2 min, followed by 30 cycles at 94°C for 30 s, 51°C for 30 s, and 68°C for 2 min 40 s. PDI C53, 397S cDNA was obtained by three rounds of PCR. In the first round of PCR, nucleotide fragment 1 was amplified with primers 1 and 2. Nucleotide fragment 2 was amplified with primers 3 and 4. In the second round of PCR, nucleotide fragment 3 was amplified with fragments 1 and 2 and primers 1 and 4. Nucleotide fragment 4 was amplified with fragment 3 and primers 1 and 5. Nucleotide fragment 5 was amplified with fragment 3 and primers 4 and 6. The third round of PCR was performed with fragments 4 and 5 and primers 1 and 4. The primers for HIF-1alpha WT cDNA in the 3×FLAG-pcDNA4 vector were primers 7 and 8. HIF-1alpha K719T was obtained by PCR with two steps. In the first round of PCR, nucleotide fragment 6 was amplified with primers 7 and 9. Nucleotide fragment 7 was amplified with primers 8 and 10. The second round of PCR was performed with fragments 6 and 7 and primers 7 and 8. Amplified PDI C53, 397S, HIF-1alpha WT, or HIF-1alpha K719T cDNA was digested with the restriction enzymes *Bam*HI and *Eco*RI (PDI C53, 397) or *Eco*RV and *Not*I (HIF-1alpha WT and HIF-1alpha K719T), respectively, and then inserted into a pcDNA3.1 (+) vector (PDI C53, 397S) or 3×FLAG-pcDNA4 vector (HIF-1alpha WT or HIF-1alpha K719T), respectively. PCR for Ero-1 WT, Ero-1 C104, 131S, Ero-1 C394, 397S, and Ero-1 (24–469 aa) cDNA was conducted with KOD plus neo DNA polymerase with denaturation at 94°C for 2 min, followed by 30 cycles at 94°C for 30 s, 53°C for 30 s, and 68°C for 1 min 30 s. The primers for Ero-1 WT cDNA were primers 11 and 12. Ero-1 C104, 131S cDNA was obtained by three rounds of PCR. In the first round of PCR, nucleotide fragment 8 was amplified with primers 11 and 13. Nucleotide fragment 9 was amplified with primers 14 and 12. In the second round of PCR, nucleotide fragment 10 was amplified with fragments 8 and 9 and primers 11 and 12. Nucleotide fragment 11 was amplified with fragment 10 and primers 11 and 15. Nucleotide fragment 12 was amplified with fragment 10 and primers 16 and 12. The third round of PCR was performed with fragments 11 and 12 and primers 11 and 12. Ero-1 C394, 397S cDNA was obtained by three rounds of PCR. In the first round of PCR, nucleotide fragment 13 was amplified with primers 11 and 17. Nucleotide fragment 14 was amplified with primers 18 and 12. In the second round of PCR, nucleotide fragment 15 was amplified with fragments 13 and 14 and primers 11 and 12. Nucleotide fragment 16 was amplified with fragment 15 and primers 11 and 19. Nucleotide fragment 17 was amplified with fragment 15 and primers 20 and 12. The third round of PCR was performed with fragments 16 and 17 and primers 11 and 12. The primers for Ero-1 (24–469 aa) cDNA were primers 21 and 22. Amplified Ero-1 WT, Ero-1 C104, 131S, Ero-1 C394, 397S, or Ero-1 (24–469 aa) cDNA was digested with the restriction enzymes *Bam*HI and *Xho*I (Ero-1 WT, Ero-1 C104, 131S, or Ero-1 C394, 397S) or *Bam*HI and *Sal*I (Ero-1 (24–469 aa)), respectively, and then inserted into a pcDNA3.1 (+) vector (Ero-1 WT, Ero-1 C104, 131S, or Ero-1 C394, 397S) or pQE-80L vector (Qiagen, Hilden, Germany) (Ero-1 (24–469 aa)), respectively. PCR for HIF-1alpha (1–245 aa), HIF-1alpha (245–826 aa), HIF-1alpha C90S (1–245 aa), HIF-1alpha C210S (1–245 aa), and HIF-1alpha C219S (1–245 aa) cDNA was performed with KOD plus neo DNA polymerase with denaturation at 94°C for 2 min, followed by 30 cycles at 94°C for 30 s, 53°C for 30 s, and 68°C for 2 min 30 s. The primers for HIF-1alpha (1–245 aa) cDNA were primers 23 and 24, primers 25 and 24, or primers 26 and 27. The primers for HIF-1alpha (245–826 aa) cDNA were primers 28 and 29. Amplified  HIF-1alpha (1–245 aa)  cDNA was digested with the restriction enzymes *Bam*HI and *Eco*RI, *Sfi*I and *Eco*RI, or *Bam*HI and *Kpn*I, and inserted into the 3×FLAG-pcDNA4 , pCMV-Myc (Takara Bio Inc, Shiga, Japan), or pQE-80L vector, respectively. Amplified HIF-1alpha (245–826 aa) cDNA was digested with the restriction enzymes *Sfi*I and *Not*I and  inserted into the pCMV-Myc vector. HIF-1alpha C90S (1–245 aa), HIF-1alpha C210S (1–245 aa), and HIF-1alpha C219S (1–245 aa) cDNA were obtained by PCR with two steps. In the first round of PCR (HIF-1alpha C90S (1–245 aa)), nucleotide fragment 18 was amplified with primers 23 and 30. Nucleotide fragment 19 was amplified with primers 31 and 24. The second round of PCR was performed with fragments 18 and 19 and primers 23 and 24. In the first round of PCR (HIF-1alpha C210S (1–245 aa)), nucleotide fragment 20 was amplified with primers 23 and 32. Nucleotide fragment 21 was amplified with primers 33 and 24. The second round of PCR was performed with fragments 20 and 21 and primers 23 and 24. In the first round of PCR (HIF-1alpha C219S (1–245 aa)), nucleotide fragment 22 was amplified with primers 23 and 34. Nucleotide fragment 23 was amplified with primers 35 and 24. The second round of PCR was performed with fragments 22 and 23 and primers 23 and 24. Amplified HIF-1alpha C90S (1–245 aa), HIF-1alpha C210S (1–245 aa), and HIF-1alpha C219S (1–245 aa) cDNA was digested with the restriction enzymes *Bam*HI and *Eco*RI, and inserted into the 3×FLAG-pcDNA4.

**Table 1:** Primers used to clone PDI C53, 397S, HIF-1alpha WT, HIF-1alpha K719T, Ero-1 WT, Ero-1 (24–469 aa), Ero-1 C104, 131S, Ero-1 C394, 397S, HIF-1alpha (1–245 aa), HIF-1alpha (245–826 aa), HIF-1alpha C90S (1–245 aa), HIF-1alpha C210S (1–245 aa), and HIF-1alpha C219S (1–245 aa).

Factor	Primer no.	Sequence	GenBank
			accession number
PDI	1	ATTGGATCCGAC**ATG**CTGCGCCGCGCTCTGCTGTGCC	NM_000918
C53, 397S	2	AGCCTTGCAGTGGCC**ACT**CCAAGGGGCATAGAA	
	3	CACTGCAAGGCTCTGGCCCCT	
	4	AATGAATTCGTA**TTA**CAGTTCATCTTTCACAGCTTTC	
	5	CAACTGTTTGCAGTGACC**ACT**CCATGGGGCATAGAA	
	6	CACTGCAAACAGTTGGCTCCC	
HIF-1alpha	7	TTGATATC**ATG**GAGGGCGCCGGCGGCGC	NM_001530
WT	8	ATAATTTAGCGGCCGC**TCA**GTTAACTTG	
HIF-1alpha	9	TTCTCTGAGCATTCTGCAAA	NM_001530
K719T	10	AGAATGCTCAGAGA**ACG**CGAAAAATGGAACA	
Ero-1 WT	11	AAGGATCC**ATG**GGCCGCGGCTGGGGATT	NM_014584
	12	TTTCTCGAG**TTA**ATGAATATTCTGTAACA	
Ero-1	13	ACTTCATCAGATTG**AGA**TGGTTTGACAGCAC	NM_014584
C104, 131S	14	TCAATCTGATGAAGTTCCTG	
	15	CGTTCAGCTTGTTC**AGA**TTCTTCAATGAGAT	
	16	TGAACAAGCTGAACGACTTG	
Ero-1	17	CAGACGACATTTAAA**AGA**ACCAACACAATCCAT	NM_014584
C394, 397S	18	AAATGTCGTCTGTGGGGAAAG	
	19	TTTCCCCACAGACG**AGA**TTTAAA**AGA**ACCAA	
	20	CTGTGGGGAAAGCTTCAGACT	
Ero-1	21	AAGGATCCGAGGAGCAGCCCCCGGAGAC	NM_014584
(24–469 aa)	22	TTAGTCGAC**TTA**ATGAATATTCTGTAACA	
HIF-1alpha	23	AAGGATCC**ATG**GAGGGCGCCGGCGGCGCGAAC	NM_001530
(1–245 aa)	24	TGAATTC**TTA**TCGACTGAGGAA	
	25	ATGGCCATGGAGGCC**ATG**GAGGGCGCCGGCGGCGCG	
	26	AAGGATCC**ATG**GAGGGCGCCGGCGGCGCGAAC	
	27	TTGGTACC**TCA**TCGACTGAGGAAAGT	
HIF-1alpha	28	ATGGCCATGGAGGCCCGACACAGCCTGGAT	NM_001530
(245–826 aa)	29	TTAGCGGCCGC**TCA**GTTAACTTGATCCAAAG	
HIF-1alpha C90S(1–245 aa)	30	GGCTTTCAAATAAAA**GCT**ATTCATCTGTGCTTT	NM_001530
	31	TATTTGAAAGCCTTGGATGGT	
HIF-1alpha C210S(1–245 aa)	32	TGGTTTCTTATACCC**ACT**CTGAGGTTGGTTACT	NM_001530
	33	TATAAGAAACCACCTATGACC	
HIF-1alpha C219S(1–245 aa)	34	ACAAATCAGCACCAA**GCT**GGTCATAGGTGGTTT	NM_001530
	35	GTGCTGATTTGTGAACCCATT	

Restriction sites were indicated as underlined, start codon (ATG) and stop codons (TTA or TCA) were indicated as bold characters, and mutant codons were indicated as shadow and bold characters.

### Knockdown experiment

si-PDI (Cat. No. SI02662100), si-HSC70 (Cat. No. SI02661477), si-PHD2 (Cat. No. SI04179196), and si-control (Cat. No. SI03650318) were purchased from Qiagen and transfected into Hep3B cells using Screen Fect A (Wako) in accordance with the manufacturer’s instructions. The target sequence for human PDI was 5′-CAGGACGGUCAUUGAUUACAA-3′, that for human HSC70 was 5′-AAGGACCUAAAUUCGUAGCAA-3′, and that for human PHD2 was 5′-ACCTTCAGATTCGGTCGGTAA -3′. Regarding the knockdown of human Ref-1 (GenBank accession number NM_001641), a specific sequence was inserted into the pBAsi-hU6 Neo vector (Takara) according to the manufacturer’s instructions. The target sequence for Ref-1 was 5′- AAGAAGCCCCAGATATACTGT-3′.

### RNA isolation and reverse transcriptase-PCR

Total RNA was extracted from Hep3B cells using ISOGEN (NIPPON GENE, Toyama, Japan) in accordance with the manufacturer’s instructions, and converted to cDNA by Revert AidTM M-MuLV Reverse transcriptase as follows: at 25°C for 15 min, 42°C for 60 min, and 70°C for 10 min. PCR was performed with Go Taq Green Master Mix (Promega) in accordance with the manufacturer’s instructions as follows: denaturation  at 94°C for 2 min and then 23 (HIF-1alpha), 21 (CA9 and Glut1, respectively), or 18 cycles (beta-actin) at 94°C for 30 s, 55°C for 30 s, and 72°C for 30 s. Primers for human HIF-1alpha were 5′-CCTAACGTGTTATCTGTCGC-3′ (forward) and 5′-GTCAGCTGTGGTAATCCACT-3′ (reverse). Primers for human CA9 (GenBank accession number NM_001216) were 5′-GCTTCCAGCTCCCGCCGCTC-3′ (forward) and 5′-CCGGGCCCTCCTCCAGAAAG-3′ (reverse). Primers for human Glut1 (GenBank accession number NM_006516) were 5′-TGCTCATCAACCGCAACGAG-3′ (forward) and 5′-GAAGGCCGTGTTGACGATAC-3′ (reverse). Primers for human beta-actin (GenBank accession number NM_001101) were 5′-CAAGAGATGGCCACGGCTGCT-3′ (forward) and 5′-TCCTTCTGCATCCTGTCGGCA-3′ (reverse).

### Preparation of antibodies

The antibodies against human Hsp90 [39], HIF-1alpha [40], PDI [41], beta-actin [42], NADPH-cytochrome P-450 reductase (NPR) [40], and rat Ref-1 [36] were prepared as previously described [43]. In the present study, we confirmed that our rat Ref-1 antibody recognized the human Ref-1 protein and, thus, used it in experiments. The pQE-80L vector including Ero-1 (24–469 aa) cDNA was transfected into *Escherichia* coli strain BL21 (Novagen, Madison, WI). Ero-1 (24–469 aa) was purified using Ni-NTA agarose (Qiagen) in accordance with the manufacturer’s instructions, and then used to preparation an antibody in rabbits using a previously described method [43]. All experiments were conducted in accordance with guidelines on the welfare of experimental animals and with the approval of the Ethics Committee on the use of animals of Kwansei Gakuin University.

### Immunoprecipitation

Hep3B cells were lysed in immunoprecipitation buffer (50 mM Tris-HCl, pH 7.5 and 150 mM NaCl) containing 0.5% Nonidet P-40 and 0.1% protease inhibitor mixture. After centrifugation at 14,000×*g* for 15 min, the supernatant was incubated with 2 μl of the anti-HIF-1alpha antibody or unimmunized rabbit serum at 4°C for 2 h. Twenty microliters of Protein A-Sepharose (50% (w/v); GE Healthcare, Chicago, IL) was added to this solution and incubated at 4°C for 1 h. Samples were washed with immunoprecipitation buffer containing 0.05% Nonidet P-40.

### Purification of Histidine (His)-tagged PDI, PDI C53, 397S, Ero-1 (24–469 aa), or HIF-1alpha (1–245 aa)

BL21 (Novagen) *E*. *coli* cells were transformed with pQE-80L encoding human PDI, PDI C53, 397S, Ero-1 (24–469 aa), or HIF-1alpha (1–245 aa). The purification of human His-tagged PDI, PDI C53, 397S, Ero-1 (24–469 aa), or HIF-1alpha (1–245 aa) was performed as described previously [44].

### Immunofluorescence assay

HEK293 cells were grown on glass-bottomed dishes (Thermo Fisher Scientific, Waltham, MA). They were then washed with PBS, fixed with 4% paraformaldehyde (Wako) at 4°C for 10 min, washed twice with TPBS (PBS containing 0.2% Tween20 (Bio-Rad, Hercules, CA)), and blocked with 0.1% bovine serum albumin (Wako) at 4°C for 1 h. Cells were then incubated with an anti-FLAG tag antibody (1:2000 dilution) at 4°C for 1 h, washed twice with TPBS, and then incubated with a daylight 594 anti-mouse secondary antibody (1:2000 dilution, Invitrogen) at 4°C for 1 h. Cells were washed twice with TPBS, and immunofluorescence was detected by confocal microscopy (Nikon A1, Tokyo, Japan). The nucleus was counterstained using DAPI (Dojindo, Kumamoto, Japan).

### Extraction of the cytosol fraction

Hep3B cells were collected with hypotonic buffer (10 mM Hepes, (pH 7.9) containing10 mM KCl, 1.5 mM MgCl_2_, 0.1% protease inhibitor mixture, 1 mM EDTA, and 0.1 mM neocuproine). Cells were homogenized and centrifuged at 1000×*g* at 4°C for 7 min to remove insoluble fractions. Lysates were centrifuged at 20000×*g* at 4°C for 30 min. Supernatants were centrifuged at 100000×*g* at 4°C for 60 min. The supernatants or pellets obtained were collected as the cytosol or microsomal fractions, respectively.

### Biotin-switch assay

Hep3B cells were solubilized in HENC buffer (HEN buffer containing 0.4% CHAPS) (HEN buffer: 250 mM Hepes, pH 7.5, 1 mM EDTA, and 0.1 mM neocuproine) on ice for 30 min, and centrifuged at 13,000×*g* at 4°C for 5 min. A one-tenth volume of 25% SDS and 20 mM MMTS were added to the supernatant and incubated at 50°C for 20 min with frequent vortexing to block free thiols. To remove excess MMTS, proteins were precipitated with 2 volumes of pre-chilled (-20°C) acetone, incubated at -20°C for 2 h, and centrifuged at 13,000×*g* for 5 min followed by gentle rinsing of the pellet 3 times with 70% acetone. The precipitate was dissolved in 0.2 ml of HENS buffer (HEN buffer containing 1% SDS), disulfide bonds in proteins were reduced by 10 mM DTT, and proteins were precipitated with acetone. The precipitate was dissolved in HENS buffer, 1 mM biotin-HPDP (prepared freshly in N,N-dimethylformamide) was added, and cells were incubated at room temperature for 1 h. Samples were precipitated with acetone and resuspended in 0.1 ml of HENS buffer. Samples were diluted in 2 volumes of neutralization buffer (20 mM HEPES, pH 7.5, 0.1 M NaCl, 1 mM EDTA, and 0.5% Triton X-100). Biotinylated proteins were incubated with 10 μl of 50% (v/v) streptavidin-agarose beads (Pierce, Rockford, IL) at room temperature for 1 h. Samples were then centrifuged at 6,000×*g* for 1 min, and the supernatant containing unbound proteins was discarded. Beads were washed 3 times with washing buffer (20 mM HEPES, pH 7.5, 600 mM NaCl, 1 mM EDTA, and 0.5% Triton X-100). To elute bound proteins, beads were incubated with 10 μl of elution buffer (20 mM HEPES, pH 7.7, 0.1 M NaCl, 1 mM EDTA, and 100 mM 2-mercaptoethanol) at 37°C for 20 min. Supernatants were collected by centrifugation, separated on SDS-PAGE, and subjected to a Western blot analysis.

### Detection of the redox state using PEG-maleimide

Hep3B cells were washed and harvested with HENC buffer, and lysates were incubated in the absence or presence of 5 mM diamide or 10 mM DTT on ice for 30 min. Proteins were precipitated with ice-cold acetone. Precipitates were dissolved in 50 mM Hepes, pH 7.5, containing 1% SDS and incubated with 20 mM NEM at room temperature for 1 h to block free thiols. Excess NEM was removed by acetone precipitation, and disulfide bonds in proteins were reduced by 1 mM DTT at room temperature for 30 min. After acetone precipitation, precipitates were dissolved in 50 mM Hepes, pH 7.5, containing 1% SDS, and then incubated with 1 mM 5 kDa PEG-maleimide for 1 h. Proteins were separated on SDS-PAGE and subjected to a Western blot analysis.

### Detection of the redox state using NEM

In total, 350 nM purified His-tagged PDI WT was added to 350 nM purified His-tagged HIF-1alpha (1–245 aa) and incubated at room temperature for 30 min. Proteins were treated with 1% SDS in the presence or absence of 10 mM DTT at room temperature for 30 min, followed by 50 mM NEM at room temperature for 1 h. Proteins were separated on non-reducing SDS-PAGE and subjected to a Western blot analysis.

### Detection of the redox state using HRP

In total, 350 nM purified His-tagged PDI WT or PDI C53, 397S was added to 350 nM purified His-tagged HIF-1alpha (1–245 aa) and incubated at room temperature for 30 min. Proteins were then treated with 1% SDS and 20 mM NEM and incubated at room temperature for 1 h. Proteins were precipitated with acetone, dissolved in HEN buffer, and incubated with 10 mM DTT at room temperature for 30 min. They were precipitated with acetone, dissolved in HEN buffer, and incubated with HRP Maleimide Conjugate at room temperature for 2 h. Proteins were separated on non-reducing SDS-PAGE and subjected to a Western blot analysis.

### Statistical analysis

Statistical analyses were performed using the Student’s *t*-test, and *p* <0.05 and *p* <0.01 were considered to be significant.

## Results

### PDI regulates the transcriptional activity and expression of HIF-1alpha

**Fig 1:**
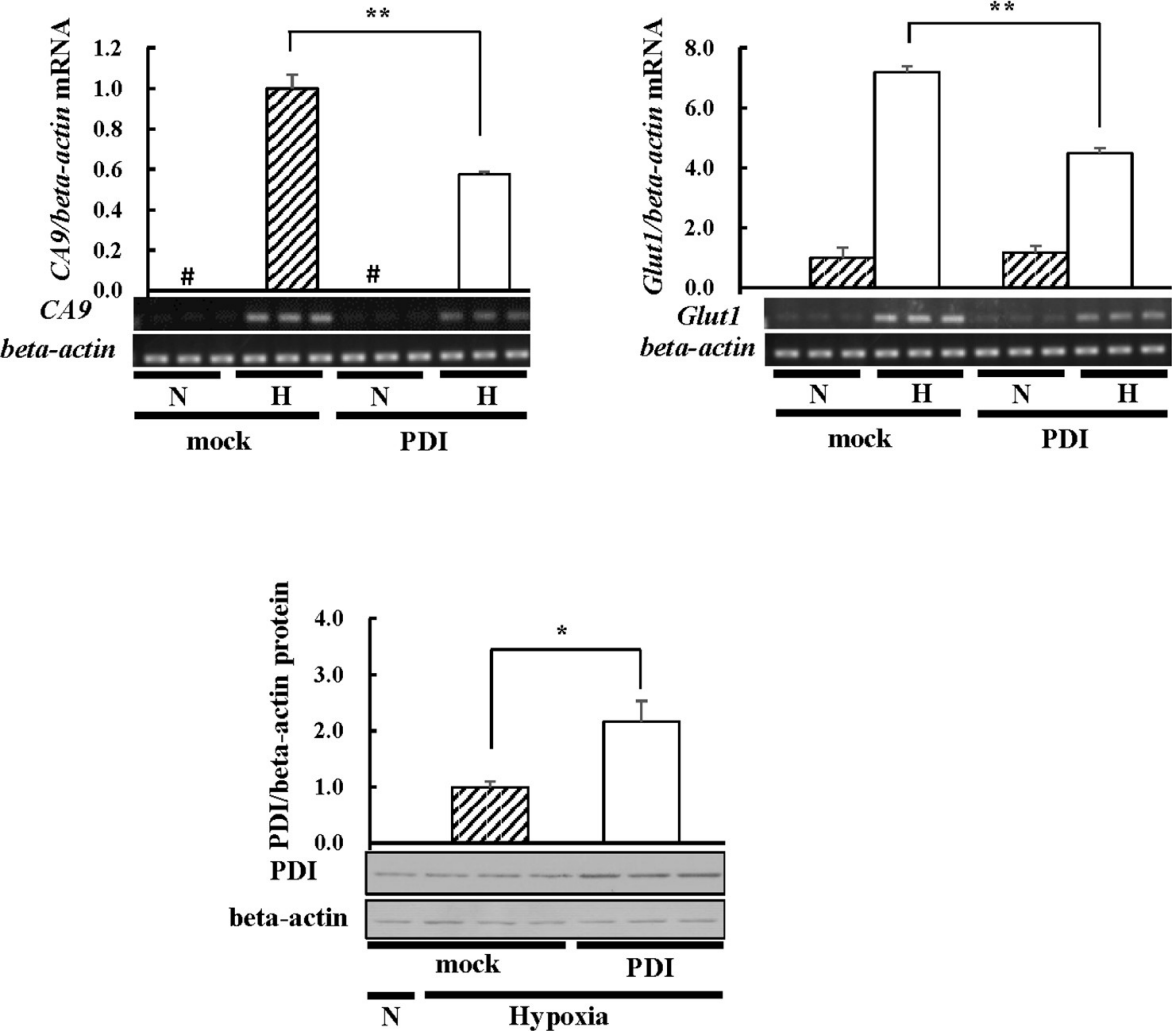
*CA9* and *Glut1* mRNA levels in PDI-overexpressing cells. Hep3B cells overexpressing PDI were cultured for 6 h under hypoxic conditions. Total RNA was isolated from cells in three different plates, and the mRNA levels of CA9 or Glut1 were assessed by RT-PCR.  The *Glut1* mRNA levels of mock cells under normoxic conditions or the *CA9* mRNA levels of mock cells under hypoxic conditions were set to 1.0. Proteins (3 μg) were separated by SDS-PAGE and immunoblotting was performed with an anti-PDI or -beta-actin antibody (1:1000 dilution). The PDI protein levels of mock cells under hypoxic conditions were set at 1.0. Values are expressed as the mean ± S.D. (*error bars*) of three different plates. N, Normoxia; H, Hypoxia. * *p*<0.05; ** *p*<0.01, significantly different from mock cells under hypoxic conditions. #, less than 0.05.

**Fig 2:**
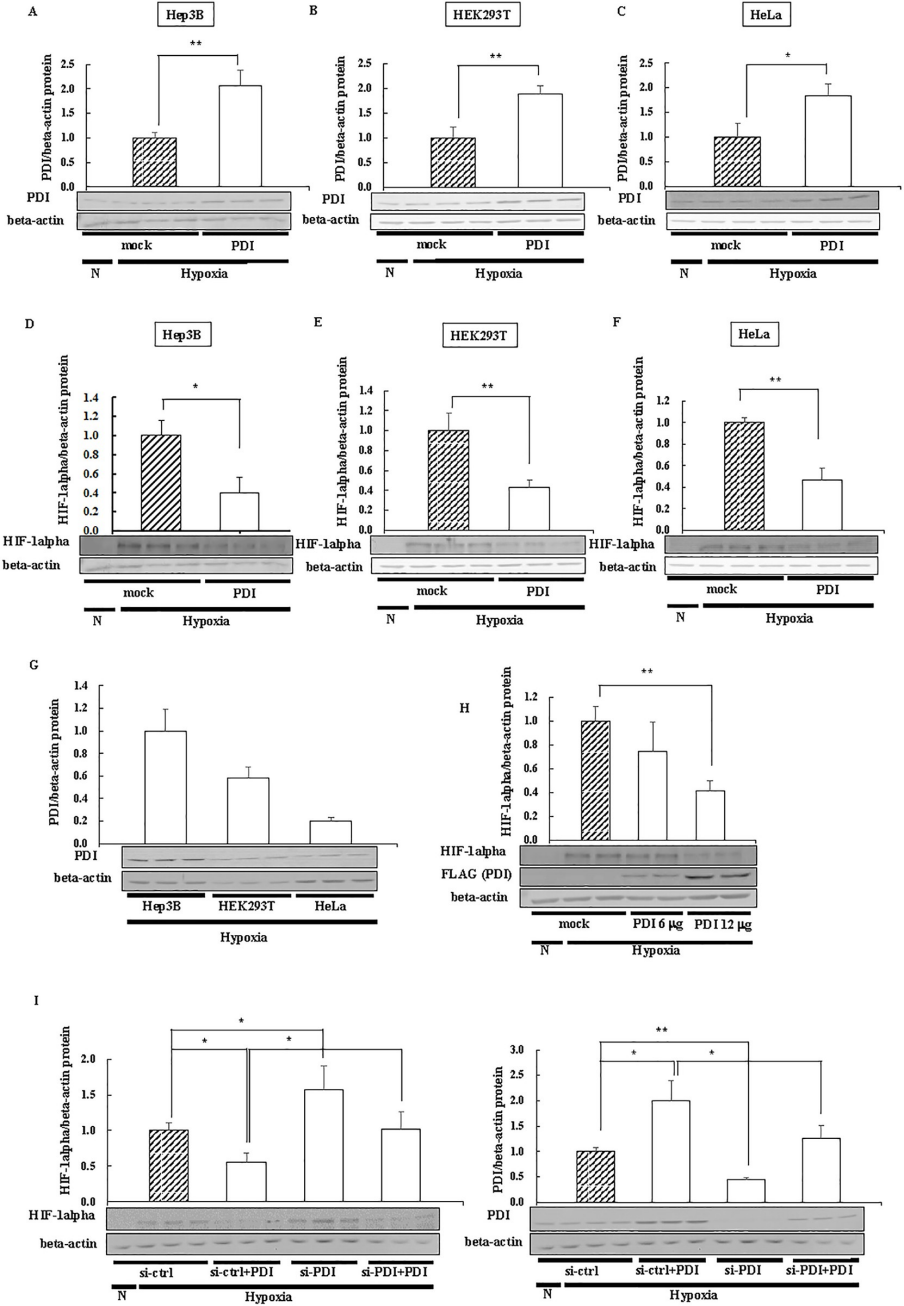
HIF-1alpha protein levels in PDI-overexpressing cells. (A-F) PDI was overexpressed in Hep3B cells (A and D), HEK293T cells (B and E), and HeLa cells (C and F), and cells were cultured for 6 h under hypoxic conditions. Proteins (3 or 15 μg) were separated by SDS-PAGE and immunoblotting was performed with an anti-PDI, -HIF-1alpha, and -beta-actin antibody (1:1000 dilution). (G) Hep3B cells, HEK293T cells, and HeLa cells were cultured for 6 h under hypoxic conditions. The expression of PDI was evaluated by immunoblotting with an anti-PDI antibody. (H) The PDI/3×FLAG-pcDNA4 vector (6 or 12 μg) was transfected into Hep3B cells, and cells were cultured for 6 h under hypoxic conditions. Proteins (3 μg) were separated by SDS-PAGE, and immunoblotting was performed with an anti-FLAG tag antibody (1:1000 dilution). (I) PDI/pcDNA 3.1 (+) and si-PDI were transfected into Hep3B cells, cells were cultured for 6 h under hypoxic conditions, and immunoblotting was performed. The PDI or HIF-1alpha protein levels of mock cells (A-F and H), or si-ctrl cells (I) under hypoxic conditions were set at 1.0. Values are expressed as the mean ± S.D. (*error bars*) of three different plates.   N, Normoxia. * *p*<0.05; ** *p*<0.01, significantly different from mock or ctrl cells (A-G) or si-ctrl cells (I) under hypoxic conditions.

PDI is up-regulated in various types of cancers [6, 7], in which a common feature is hypoxia. Although HIF-1alpha is a key factor in hypoxia, the relationship between HIF-1alpha and PDI currently remains unclear; therefore, we investigated the effects of PDI on HIF-1alpha using the human hepatocarcinoma cell line, Hep3B. PDI was overexpressed in Hep3B cells, and PDI-overexpressing cells were cultured under hypoxic conditions. Since PDI activates several transcription factors [34], we investigated the effects of PDI on the transcriptional activity of HIF-1alpha. *CA9* and *Glut1* mRNA levels were elevated under hypoxic conditions and decreased by PDI (Fig 1). The rate of increases in PDI expression were correlated with the rate of decreases in *CA9* and *Glut1* mRNA levels. These results were contradictory to previous findings. Therefore, we investigated whether the effects of PDI on HIF-1alpha were dependent on protein levels. We confirmed the overexpression of PDI in each cell (Fig 2A–2C). The rate of increases in PDI expression after overexpression of PDI in three kinds of cells was approximately the same. The results obtained showed that PDI decreased HIF-1alpha expression levels in Hep3B cells (Fig 2D). We obtained similar results from HEK293T cells (Fig 2E) and HeLa cells (Fig 2F), suggesting that this phenomenon also occurred in normal cells and was independent of the cell type. Moreover, the rate of increases in PDI expression were correlated with the rate of decreases in HIF-1alpha expression in each cell (Fig 2A–2F). Hep3B cells have the highest expression of PDI of three kinds of cells (Fig 2G). Hep3B cells may have the highest susceptibility to therapy targeting PDI and show the strongest effect of endogenous PDI on HIF-1alpha. In subsequent experiments, different amounts of PDI containing the FLAG tag were overexpressed in Hep3B cells. HIF-1alpha expression decreased in a dose-dependent manner (Fig 2H). Therefore, we prepared PDI-knockdown cells by si-RNA and investigated the effects of the knockdown of PDI on endogenous HIF-1alpha expression. The knockdown of endogenous PDI by si-PDI increased HIF-1alpha expression levels, and the decreases in HIF-1alpha expression levels caused by the overexpression of PDI was restored by the co-transfection of si-PDI with PDI/pcDNA 3.1 (+) (Fig 2I). The rate of change in PDI expression were approximately correlated with the rate of change in HIF-1alpha expression. These results indicated that the expression of HIF-1alpha was dependent on PDI expression levels.

### PDI decreases HIF-1alpha expression levels via its redox activity

**Fig 3:**
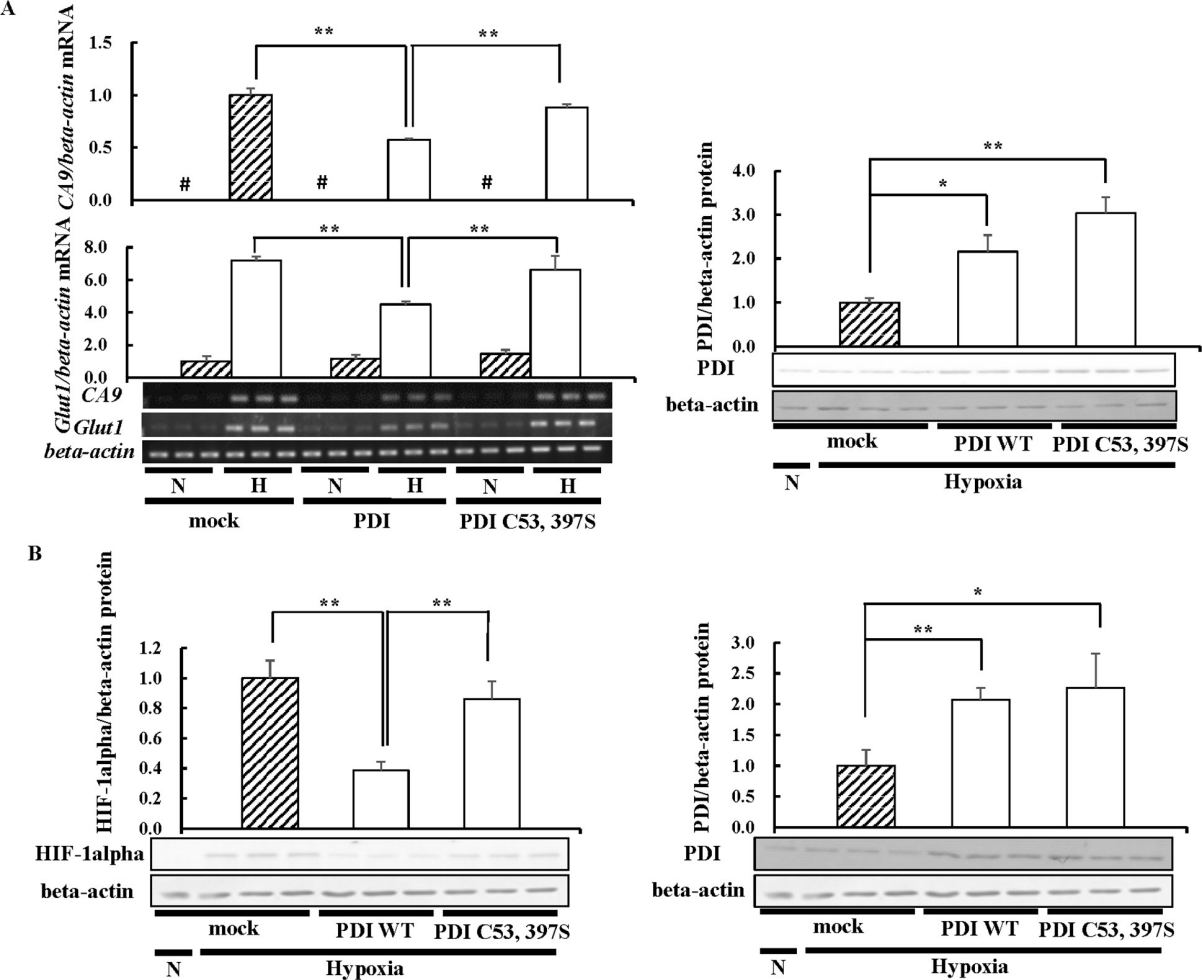
HIF-1alpha protein levels in catalytic inactive mutant PDI-overexpressing cells. (A) Hep3B cells overexpressing PDI WT or PDI C53, 397S were cultured for 6 h under hypoxic conditions, and the mRNA levels of CA9 or Glut1 were assessed by RT-PCR. (B) Hep3B cells overexpressing PDI WT or PDI C53, 397S were cultured for 6 h under hypoxic conditions and then subjected to immunoblotting. The overexpression of PDI WT or PDI C53, 397S in Hep3B cells was assessed by Western blotting with an anti-PDI antibody (*right panel*) (A and B). The HIF-1alpha or PDI protein levels of mock cells under hypoxic conditions were set to 1.0. Values are expressed as the mean ± S.D. (*error bars*) of three different plates. N, Normoxia; H, Hypoxia. * *p*<0.05; ** *p*<0.01, significantly different from mock cells under hypoxic conditions.  #, less than 0.05.

PDI consists of the a, b, b’, and a’ domains and has a TRX-like motif, a catalytic active site, in the a and a’ domains [45]. These catalytic sites have two cysteine residues, respectively, and mutations in these cysteine residues result in the loss of redox activity. We previously reported that PDI suppresses growth hormone (GH) expression, whereas the PDI mutant does not [36]. Therefore, we examined whether the decrease observed in HIF-1alpha expression levels by PDI occurred via its redox activity. We prepared a mutant of PDI, designated PDI C53, 397S, in which the 53rd and 397th cysteine residues were replaced by serine residues, and PDI C53, 397S was overexpressed in Hep3B cells. The results obtained showed that the overexpression of PDI WT decreased *CA9* and *Glut1* mRNA levels (Fig 3A). We also investigated the effects of the redox activity of PDI on HIF-1alpha expression. The overexpression of PDI WT decreased HIF-1alpha expression levels, whereas the overexpression of PDI C53, 397S did not (Fig 3B). The increases in PDI C53, 397S expression levels were the same or higher than those in PDI WT (Fig 3A and 3B). These results suggested that the redox activity of PDI plays a critical role in the decreases observed in HIF-1alpha expression levels.

### Effects of Ero-1 on oxidized PDI and HIF-1alpha expression

**Fig 4:**
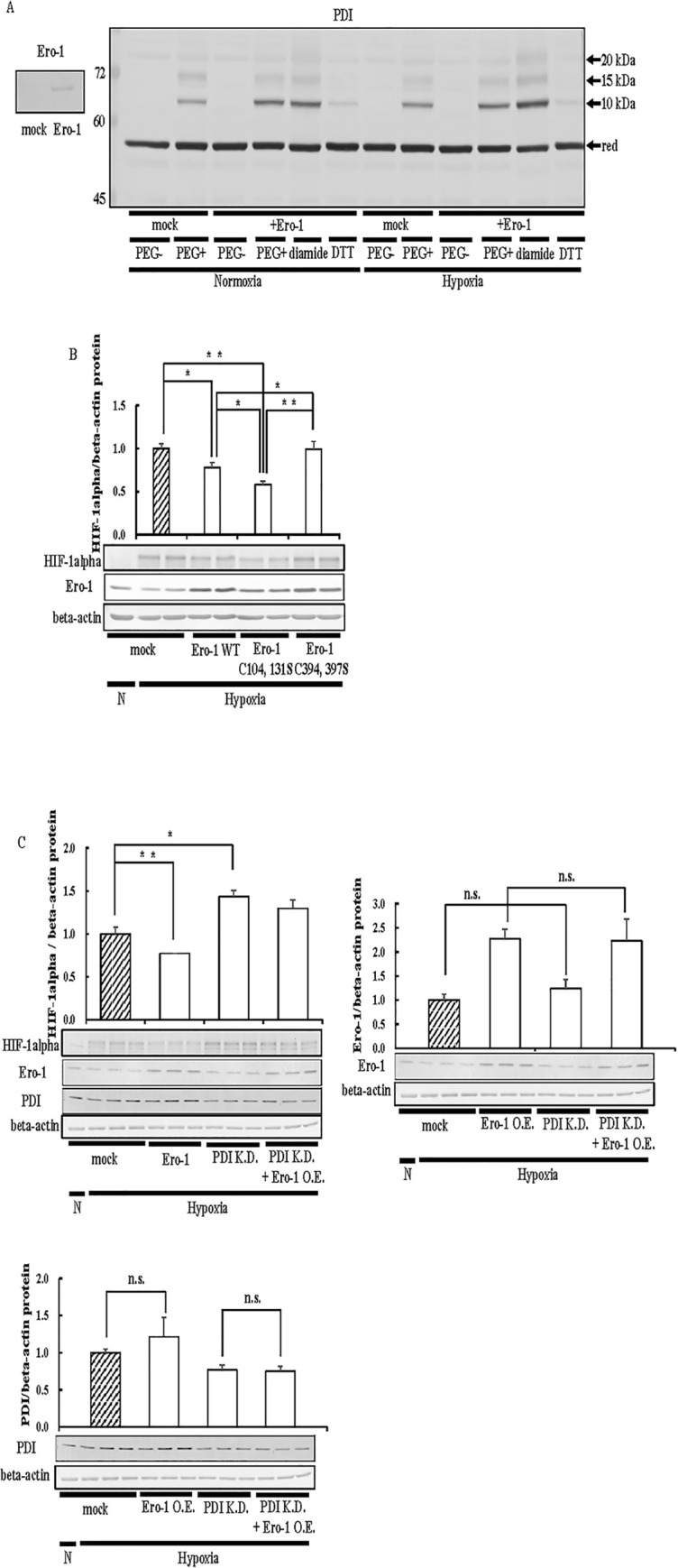
Effects of Ero-1 on HIF-1alpha expression. (A) Hep3B cells overexpressing Ero-1 WT were cultured for 6 h under hypoxic conditions. Cells were harvested with PBS, and proteins were incubated in the absence or presence of 5 mM diamide or 10 mM DTT. Precipitated proteins were incubated with 20 mM NEM. After the removal of NEM, proteins were incubated with 1 mM DTT. Precipitated proteins were incubated with 1 mM 5kDa PEG-maleimide. An up-shift in molecular weight by the binding of PEG-maleimide was detected by immunoblotting with the anti-PDI antibody. To evaluation of the overexpression of Ero-1 (*left panel*), proteins (3 μg) were separated by SDS-PAGE, and immunoblotting was performed with the anti-Ero-1 antibody (1:1000 dilution). red, Reduced form. (B) Hep3B cells overexpressing Ero-1 WT or Ero-1 C104, 131S, and Ero-1 C394, 397S were cultured for 6 h under hypoxic conditions, and immunoblotting was performed. (C) Ero-1/pcDNA 3.1 (+) and si-PDI were transfected into Hep3B cells, cells were cultured for 6 h under hypoxic conditions, and immunoblotting was performed. The overexpression of Ero-1 or PDI was evaluated by immunoblotting with an anti-Ero-1 or PDI antibody (1:1000 dilution). The HIF-1alpha, Ero-1, or PDI protein levels of mock cells under hypoxic conditions were set to 1.0. Values are expressed as the mean ± S.D. (*error bars*) of three different plates. N, Normoxia; H, Hypoxia; n. s., not significant. * *p*<0.05; ** *p*<0.01, significantly different from   mock cells under hypoxic conditions.

Since HIF-1alpha expression levels were decreased by the redox activity of overexpressed PDI, we investigated whether changes in the redox state of endogenous PDI affect HIF-1alpha expression levels. PDI is oxidized (produces a disulfide bond in PDI) by endoplasmic reticulum oxidoreductin-1 (Ero-1) [46]. Furthermore, the expression of Ero-1 and PDI is up-regulated in cancer [47, 48]. Therefore, we examined the effects of the oxidation of endogenous PDI by Ero-1 on HIF-1alpha expression levels. Since Ero-1 requires molecular oxygen to oxidize PDI [46], we initially investigated whether Ero-1 oxidized PDI under hypoxic conditions. We detected the PDI redox state in Ero-1-overexpressing cells using 5-kDa PEG-maleimide. We detected up-shifts in the PDI band in mock cells by 10 and 20 kDa. Up-shifted bands at the same mobility were detected in cells treated with diamide, and these bands disappeared following the treatment with DTT (Fig 4A). From these results, we confirmed that the oxidized form of PDI was detected as an up-shifted band. The intensities of the up-shifted bands of PDI were increased by the overexpression of Ero-1 under normoxic conditions, and similar results were obtained under hypoxic conditions (Fig 4A). These results indicated that Ero-1 increased the oxidized form of PDI under hypoxic conditions. An up-shift in the PDI band by 15 kDa may appear with the formation of a heterodimer between PDI and Ero-1 [49]. We then investigated the effects of an increase in the oxidized form of PDI on HIF-1alpha expression. The overexpression of Ero-1 WT decreased HIF-1alpha expression levels (Fig 4B). We then prepared redox isoforms of Ero-1. The oxidation activity of Ero-1 is known to be dependent on disulfide bond rearrangements [50]. The overexpression of Ero-1 C104, 131S, which is an enhancer form of Ero-1 activity, decreased HIF-1alpha expression levels more significantly than Ero-1 WT (Fig 4B). The overexpression of Ero-1 C394, 397S, which does not exhibit oxidative activity, did not affect HIF-1alpha expression levels (Fig 4B). These results suggested that although the increase observed in the oxidized form of PDI was important for decreasing HIF-1alpha expression levels, we cannot exclude the possibility that Ero-1 directly reduced these levels. To confirm the involvement of PDI in the degradation of HIF-1alpha by Ero-1, we prepared PDI-knockdown cells. Ero-1 did not decrease HIF-1alpha expression levels in PDI-knockdown cells (Fig 4C). Moreover, overexpression of Ero-1 did not affect PDI expression (Fig 4C). Knockdown of PDI did not affect Ero-1 expression (Fig 4C). These results indicated that the oxidation activity of Ero-1 down-regulated HIF-1alpha expression via PDI.

### Effects of PHD on decreases in HIF-1alpha expression levels by PDI

**Fig 5:**
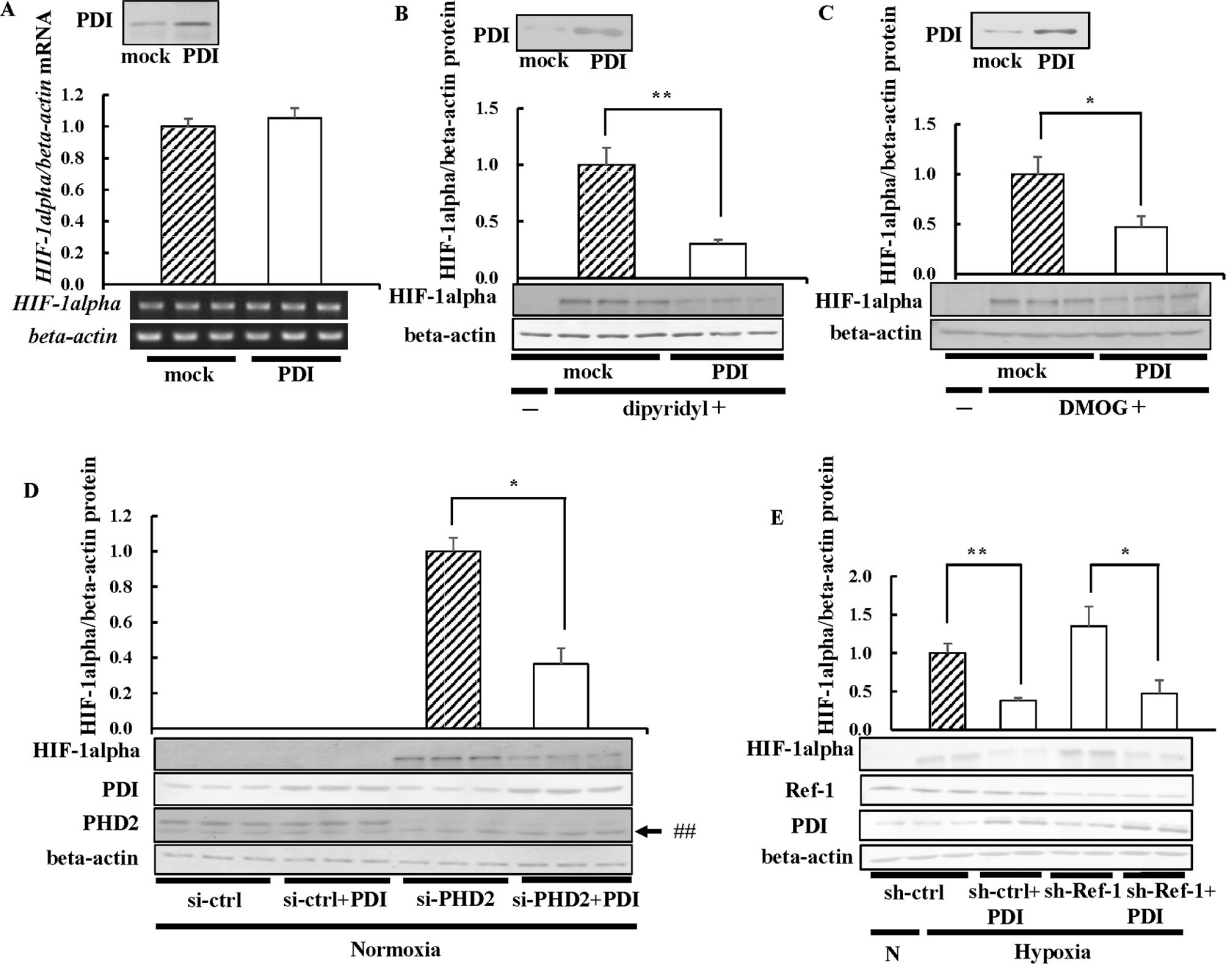
Effects of PHD on HIF-1alpha expression. (A) Hep3B cells overexpressing PDI were cultured under normoxic conditions, and total RNA was isolated from cells in three different plates. HIF-1alpha mRNA levels were measured by RT-PCR. The *HIF-1alpha* mRNA levels of mock cells were set to 1.0. (B and C) Hep3B cells overexpressing PDI were cultured for 6 h in the presence of 100 μM dipyridyl (B) or for 16 h in the presence of 0.5 mM DMOG (C) under normoxic conditions, and immunoblotting was performed. The HIF-1alpha protein levels of mock cells in the presence of dipyridyl (B) or DMOG (C) were set to 1.0. (D and E) PDI/pcDNA 3.1 (+) and si-PHD2 (D) or sh-Ref-1 (E) were transfected into Hep3B cells, cells were cultured for 6 h under hypoxic conditions (E). Proteins (3 or 15 μg) were separated by SDS-PAGE, and immunoblotting was performed with the anti-PHD2 or -Ref-1 antibody (1:1000 dilution). The HIF-1alpha protein levels of si-ctrl or sh-ctrl cells under hypoxic or normoxia conditions were set to 1.0. Values were expressed as the mean ± S.D. (*error bars*) of three different plates. N, Normoxia; ctrl, control; ##, non-specific band. * *p*<0.05; ** *p*<0.01, significantly different from   mock cells in the presence of dipyridyl or DMOG or si-ctrl cells.

We examined whether the decrease in HIF-1alpha expression levels by PDI was associated with reductions in *HIF-1alpha* mRNA levels. PDI did not affect *HIF-1alpha* mRNA levels (Fig 5A), suggesting that it regulated HIF-1alpha at the protein level. HIF-1alpha is known to be decreased by PHD under normoxic conditions [1]. PHD requires molecular oxygen, ferrous iron, ascorbic acid, and alpha-ketoglutaric acid for its hydroxylation activity [51]. To establish whether PHD is involved in the regulation of HIF-1alpha expression by PDI, PDI-overexpressing cells were treated with dipyridyl, a ferrous iron chelator, or DMOG, an analogue of alpha-ketoglutaric acid, under normoxic conditions. HIF-1alpha was stabilized under normoxic conditions in the presence of dipyridyl, and even under these conditions, HIF-1alpha expression levels were decreased by the overexpression of PDI (Fig 5B). Similar results were obtained in the presence of DMOG (Fig 5C). In subsequent experiments, we prepared PHD2-knockdown cells by si-PHD2 because PHD2 is the most important among the three PHD isoforms involved in the regulation of HIF-1alpha [52]. The knockdown of endogenous PHD2 by si-PHD2 stabilized HIF-1alpha under normoxic conditions (Fig 5D). The overexpression of PDI decreased even HIF-1alpha increased by the knockdown of PHD2 under normoxic conditions (Fig 5D). These results suggested that PDI decreased HIF-1alpha expression levels in a PHD-independent manner. Ref-1 has been shown to enhance the transcriptional activity of TR [36]. We previously reported that the overexpression of PDI decreases the transcriptional activity of TR in GH3 cells by oxidizing Ref-1 [36]. Ref-1 is also shown to regulate the transcriptional activity of HIF-1alpha by reduction [37]. Therefore, we investigated the involvement of Ref-1 in decreases in HIF-1alpha expression levels by PDI. The overexpression of PDI decreased HIF-1alpha expression levels, even in Ref-1-knockdown cells (Fig 5E), suggesting that Ref-1 was not required for the decreases observed in HIF-1alpha expression levels by PDI.

### Effects of HSC70 on HIF-1alpha regulation by PDI

**Fig 6:**
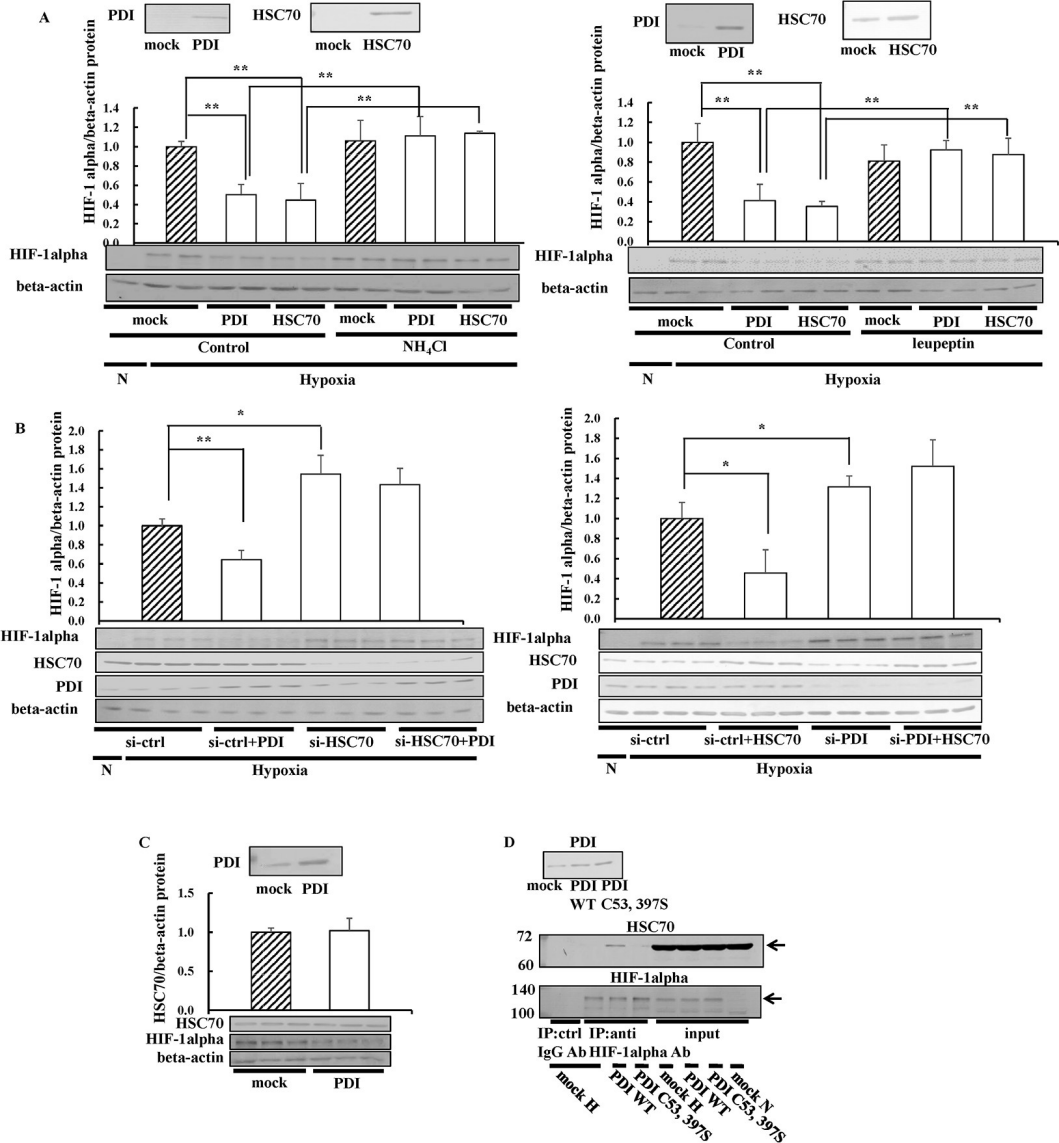
Effects of HSC70 on HIF-1alpha regulation by PDI. (A) Hep3B cells overexpressing PDI or HSC70 were cultured in the presence of 10 mM NH_4_Cl or 10 μM leupeptin for 8 h under hypoxic conditions. Proteins (3 μg) were separated by SDS-PAGE, and immunoblotting was performed with the anti-HSC70 antibody (1:1000 dilution). The overexpression of HSC70 was assessed by immunoblotting with the anti-HSC70 antibody (*upper panel*). The HIF-1alpha protein levels of mock cells in the absence of NH_4_Cl under hypoxic conditions were set to 1.0. (B) PDI/pcDNA 3.1 (+) and si-HSC70 or si-PDI and HSC70/pcDNA 3.1 (+) were transfected into Hep3B cells, cells were cultured for 6 h under hypoxic conditions, and immunoblotting was performed. The HIF-1alpha protein levels of si-ctrl cells were set to 1.0. (C) Hep3B cells overexpressing PDI were cultured for 6 h under hypoxic conditions and then subjected to immunoblotting. Values are expressed as the mean ± S.D. (*error bars*) of three different plates. N, Normoxia. * *p*<0.05, ** *p*<0.01, significantly different from   mock cells (A and C) or si-ctrl cells (B) under hypoxic conditions.  (D) Hep3B cells overexpressing PDI WT or PDI C53, 397S were cultured under hypoxic conditions for 8 h in the presence of NH_4_Cl. Cell extracts were subjected to immunoprecipitation (IP) using the anti-HIF-1alpha antibody (Ab). Precipitated proteins were separated by SDS-PAGE, and immunoblotting was performed with the anti-HSC70 or -HIF-1alpha Ab, respectively. Arrows indicate bands corresponding to HSC70 or HIF-1alpha. N, Normoxia; H, Hypoxia; ctrl, control.

Besides PHD, HIF-1alpha expression is regulated by heat shock cognate 70 kDa protein (HSC70) and lysosome-associated membrane protein type 2A (LAMP2A) in a lysosome-dependent manner [53]. HSC70 interacts with proteins that have a KFERQ-like motif and transports them to lysosomes. A non-canonical KFERQ-like motif is presented in HIF-1alpha, and, thus, HSC70 recognizes and binds to HIF-1alpha [54]. The decrease in HIF-1alpha expression levels by PDI was restored in the presence of NH_4_Cl, suggesting that PDI induced the lysosomal degradation of HIF-1alpha. The overexpression of HSC70 also decreased endogenous HIF-1alpha expression levels, which were completely restored in the presence of NH_4_Cl (Fig 6A). Similar results were obtained in the presence of leupeptin, which is an inhibitor of cathepsin B, a lysosomal protease (Fig 6A). From these results, we confirmed that HIF-1alpha was degraded in a lysosome-dependent manner and that NH_4_Cl inhibited the lysosomal pathway. To investigate the involvement of HSC70 in the decreases observed in HIF-1alpha expression levels by PDI, we prepared HSC70-knockdown cells using si-HSC70. PDI did not decrease HIF-1alpha expression in HSC70-knockdown cells (Fig 6B), suggesting that PDI induced the lysosomal degradation of HIF-1alpha via HSC70. In subsequent experiments, we investigated the effects of HSC70 overexpression on HIF-1alpha expression increased by the knockdown of PDI. The overexpression of HSC70 did not decrease HIF-1alpha expression in PDI-knockdown cells (Fig 6B), suggesting that PDI was required for the degradation of HIF-1alpha by HSC70. We speculated that PDI may degrade HIF-1alpha by increasing HSC70 expression levels. Therefore, we examined the effects of PDI on HSC70 expression, and showed that PDI did not induce any changes (Fig 6C). These results indicated that the decreases observed in HIF-1alpha expression levels by PDI were not due to the increase in HSC70 levels. Therefore, we hypothesized that PDI may affect the interaction between HIF-1alpha and HSC70. To assess the effects of PDI on the interaction between HIF-1alpha and HSC70, immunoprecipitation was performed using the antibody against HIF-1alpha in the presence of NH_4_Cl. Endogenous HSC70 immunoprecipitated with HIF-1alpha was increased by the overexpression of PDI, whereas the PDI C53, 397S mutant did not exert this effect (Fig 6D). These results suggested that PDI facilitated the binding of HSC70 with HIF-1alpha by its redox activity, leading to the lysosomal degradation of HIF-1alpha via HSC70.

### Interaction between PDI and HIF-1alpha

**Fig 7:**
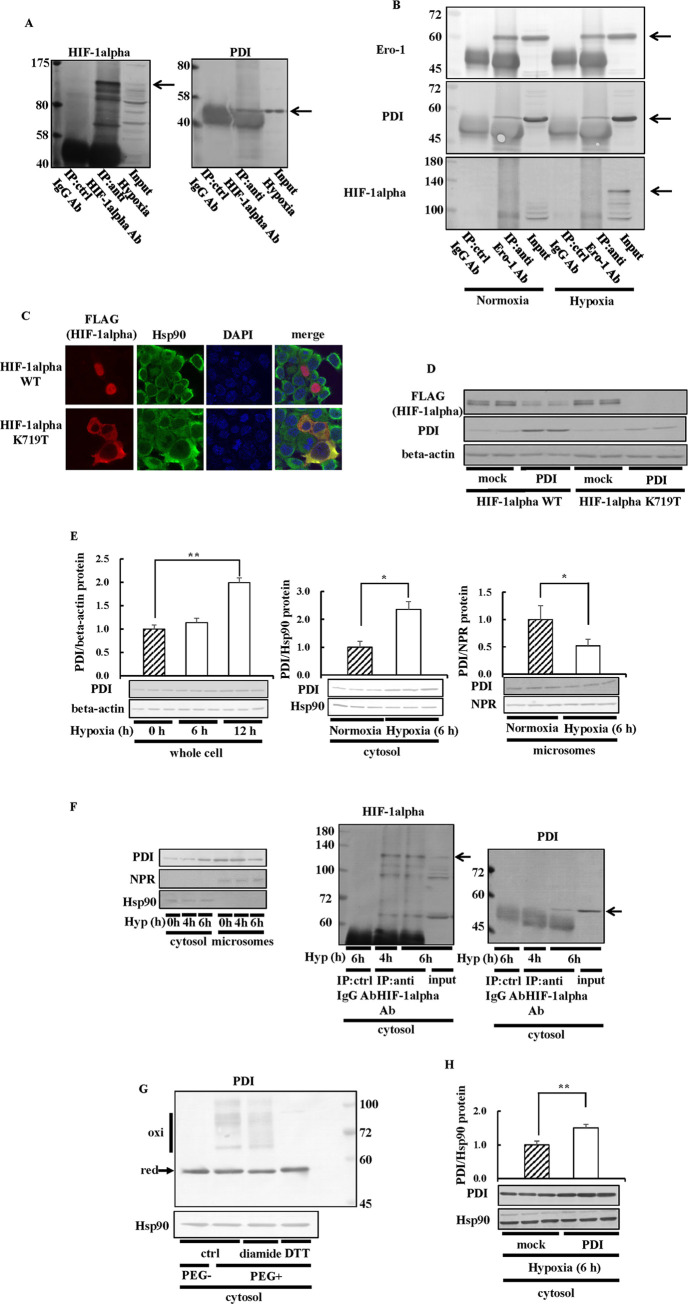
Interaction between PDI and HIF-1alpha. (A and B) Hep3B cells were cultured under hypoxic conditions for 6 h. Cell extracts were subjected to immunoprecipitation using the anti-HIF-1alpha antibody. Precipitated proteins were separated by SDS-PAGE, and immunoblotting was performed with the anti-HIF-1alpha, -PDI (A), or -Ero-1 antibody (B). Arrows indicate bands corresponding to HIF-1alpha, PDI (A and B), or Ero-1 (B). ctrl, control. (C) HEK293 cells overexpressing HIF-1alpha WT or HIF-1alpha K719T were cultured under hypoxic conditions for 6 h. Cells were stained with the anti-FLAG tag and Hsp90 antibody and analyzed under a confocal microscope. (D) HEK293 cells overexpressing HIF-1alpha WT or HIF-1alpha K719T were cultured under hypoxic conditions for 6 h, and immunoblotting was performed. Proteins (15 μg) were separated by SDS-PAGE, and immunoblotting was performed with the anti-FLAG antibody. (E) Hep3B cells were cultured under hypoxic conditions for 6 h or 12 h, and the cytosol or microsomal fraction was extracted (6 h). Proteins was separated by SDS-PAGE, and immunoblotting was performed with the anti-PDI, -NPR, or -Hsp90 antibody. (F and G) Hep3B cells were cultured under hypoxic conditions for 4 h (F) or 6 h (F and G). Cells were treated with 20 mM NEM for 15 min (G). The cytosol (F and G) or microsomal fraction (F) was extracted. Proteins was separated by SDS-PAGE, and immunoblotting was performed with the anti-PDI (F), -NPR (F), or -Hsp90 (F and G) antibody. Immunoprecipitation was performed with the anti-HIF-1alpha antibody (F). The redox states of proteins were detected using the same method as in Fig 4A (G). An up-shift in molecular weight by the binding of PEG-maleimide was detected by immunoblotting with the anti-PDI (G). (H) Hep3B cells overexpressing PDI were cultured under hypoxic conditions for 6 h, and the cytosol fraction was extracted. Proteins was separated by SDS-PAGE, and immunoblotting was performed with the anti-PDI, or -Hsp90 antibody. The PDI protein levels of ctrl, or mock cells under normoxic or hypoxic conditions were set to 1.0. Values are expressed as the mean ± S.D. (*error bars*) of three different plates. ctrl, control. * *p*<0.05; ** *p*<0.01, significantly different from ctrl, mock, or si-ctrl cells.

We hypothesized that PDI may change the redox state of HIF-1alpha and then enhance the interaction between HIF-1alpha and HSC70. Therefore, we initially investigated whether PDI directly interacts with HIF-1alpha. Immunoprecipitation was conducted using the HIF-1alpha antibody (Fig 7A). When HIF-1alpha was immunoprecipitated, PDI co-precipitated with HIF-1alpha. We found that Ero-1 decreased HIF-1alpha expression levels via PDI (Fig 4C). We also examined the relationship between HIF-1alpha and Ero-1. HIF-1alpha was not identified when Ero-1 was immunoprecipitated; however, PDI was detected by Western blotting (Fig 7B), suggesting that Ero-1 interacted with PDI, but did not interact directly with HIF-1alpha. PDI mainly exists in ER and has also been observed in the cytosol and nucleus [33]. To examine the interaction between HIF-1alpha and PDI in more detail, we prepared an HIF-1alpha mutant containing a FLAG tag, designated HIF-1alpha K719T, in which the 719th lysine in the C-terminal nuclear localization signal was replaced by threonine [55]. HIF-1alpha K719T localizes to the cytosol [55]. We confirmed that HIF-1alpha WT localized to the nucleus and HIF-1alpha K719T to the cytosol (Fig 7C). HIF-1alpha WT and the K719T mutant were both overexpressed together with PDI in HEK293 cells. As a result, PDI overexpression decreased HIF-1alpha K719T expression levels more efficiently than HIF-1alpha WT expression (Fig 7D), indicating that PDI affected HIF-1alpha expression levels in the cytosol. We speculated that the release of PDI from the ER to the cytosol is critical for the degradation of HIF-1alpha. Hypoxia is a common feature of cancer. Therefore, we investigated the effects of hypoxia on the localization of PDI (Fig 7E). The amount of PDI in total cell fraction did not change after 6 h under hypoxic conditions, whereas they increased after 12 h (Fig 7E). We indicated that the knockdown of PDI increased HIF-1alpha expression after 6 h under hypoxic conditions (Fig 2I). Therefore, we investigated the effects of hypoxia on the localization of PDI after 6 h under hypoxic conditions. The results obtained showed that the amount of PDI in the cytosol increased after 6 h under hypoxic conditions (Fig 7E). The amount of PDI in the microsomes decreased after 6 h under hypoxic conditions (Fig 7E). These results indicated that PDI was released from the ER to the cytosol by hypoxic stimulus. To investigate the effects of PDI on HIF-1alpha expression in the cytosol in more detail, we investigated the interaction between constitutive PDI and HIF-1alpha using the cytosol fraction (Fig 7F). We confirmed that NPR, a microsomes marker, was not detected in the cytosol fraction (Fig 7F). When cytosolic PDI was increased by hypoxia stimulus after 6 hours, PDI co-precipitated with HIF-1alpha in the cytosol. These results indicated that cytosolic PDI increased by hypoxic stimulus interacted with HIF-1alpha. We indicated that the increases in the oxidized form of PDI was important for the degradation of HIF-1alpha (Fig 4B). Therefore, we investigated the redox state of PDI in the cytosol (Fig 7G). We detected the oxidized form of PDI in the cytosol (Fig 7G). The overexpression of PDI increased the cytosolic PDI (Fig 7H). Collectively, these results suggested that hypoxia increased PDI expression levels in the cytosol, which resulted in the degradation of HIF-1alpha.

### PDI changes the HIF-1alpha redox state

**Fig 8:**
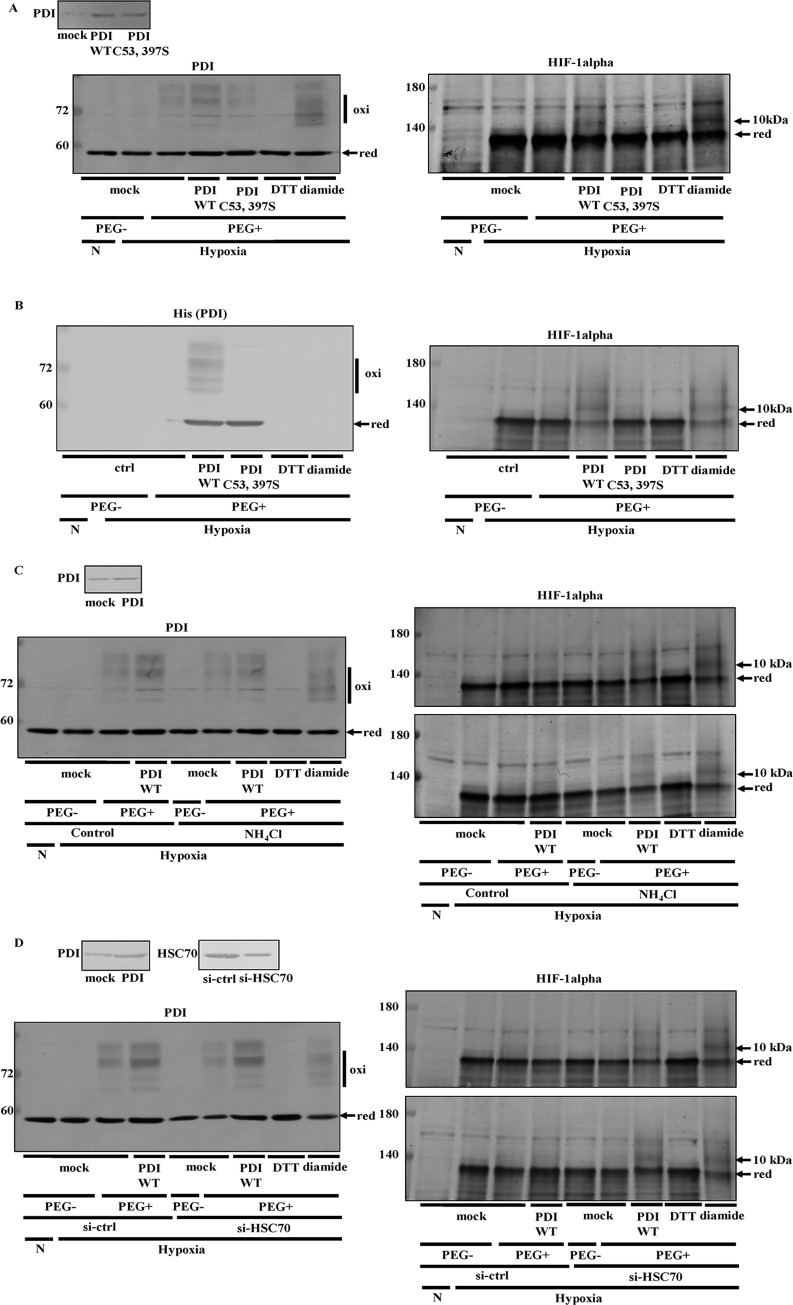
Detection of the HIF-1alpha redox state by PEG-maleimide. (A) Hep3B cells overexpressing PDI or PDI C53, 397S were cultured under hypoxic conditions for 8 h in the presence of NH_4_Cl. (B) Hep3B cells were cultured under hypoxic conditions for 6 h and harvested with 50 mM Hepes, pH 7.5, containing 0.5% Nonidet P-40. The lysate from Hep3B cells was then treated with 17.5 nM purified His-tagged PDI WT or PDI C53, 397S. The redox states of proteins were detected using the same method as in Fig 4A. An up-shift in molecular weight by the binding of PEG-maleimide was detected by immunoblotting with the anti-PDI (A), -HIF-1alpha (A and B), or -His tag antibody (B). (C and D) PDI-overexpressing Hep3B cells were cultured for 8 h under hypoxic conditions in the presence of NH_4_Cl (C). PDI/pcDNA 3.1 (+) and si-HSC70 were transfected into Hep3B cells, and cells were cultured for 6 h under hypoxic conditions (D). Cells were harvested with PBS. The redox states of proteins were detected using the same method as that in Fig 4A. Since PDI decreased HIF-1alpha expression levels in the absence of NH_4_Cl (C) or in si-ctrl cells (D), 1.5-fold amount of protein was applied in Lane 4 in order to achieve an equal intensity in the reduced form of HIF-1alpha in other lanes. The knockdown of HSC70 was checked by immunoblotting with the anti-HSC70 antibody (*upper panel*).

We analyzed the effects of PDI on the HIF-1alpha redox state. We assessed the HIF-1alpha redox state in PDI-overexpressing cells using 5-kDa PEG-maleimide (Fig 8A). An up-shift in the HIF-1alpha band by 10 kDa was detected following the treatment of cells with diamide, while this band was absent in cells treated with DTT. We detected one HIF-1alpha band that was up-shifted by 10 kDa in PDI WT-overexpressing cells, but not in PDI C53, 397S-overexpressing cells in the presence of NH_4_Cl (Fig 8A), suggesting that one disulfide bond of HIF-1alpha was formed by PDI via its redox activity. We also performed an *in vitro* experiment. We added purified His-tagged PDI WT or PDI C53, 397S to the Hep3B cell lysate. We detected one band of HIF-1alpha up-shifted by 10 kDa following the addition of purified His-tagged PDI WT to the cell lysate, but not PDI C53, 397S (Fig 8B). This result indicated that PDI directly formed a disulfide bond in HIF-1alpha. We then investigated whether oxidized HIF-1alpha was degraded via the lysosomal pathway. In the presence of NH_4_Cl, the band of HIF-1alpha oxidized by PDI (the band up-shifted by PEG-maleimide) was detected, whereas the up-shifted band disappeared in the absence of NH_4_Cl (Fig 8C), suggesting that oxidized HIF-1alpha was degraded via the lysosomal pathway. Furthermore, even in the absence of NH_4_Cl, the knockdown of HSC70 recovered oxidized HIF-1alpha (Fig 8D). Collectively, these results showed that HIF-1alpha oxidized by PDI was degraded via the lysosomal pathway with HSC70.

### Detection of the HIF-1alpha (1–245 aa) redox state

**Fig 9:**
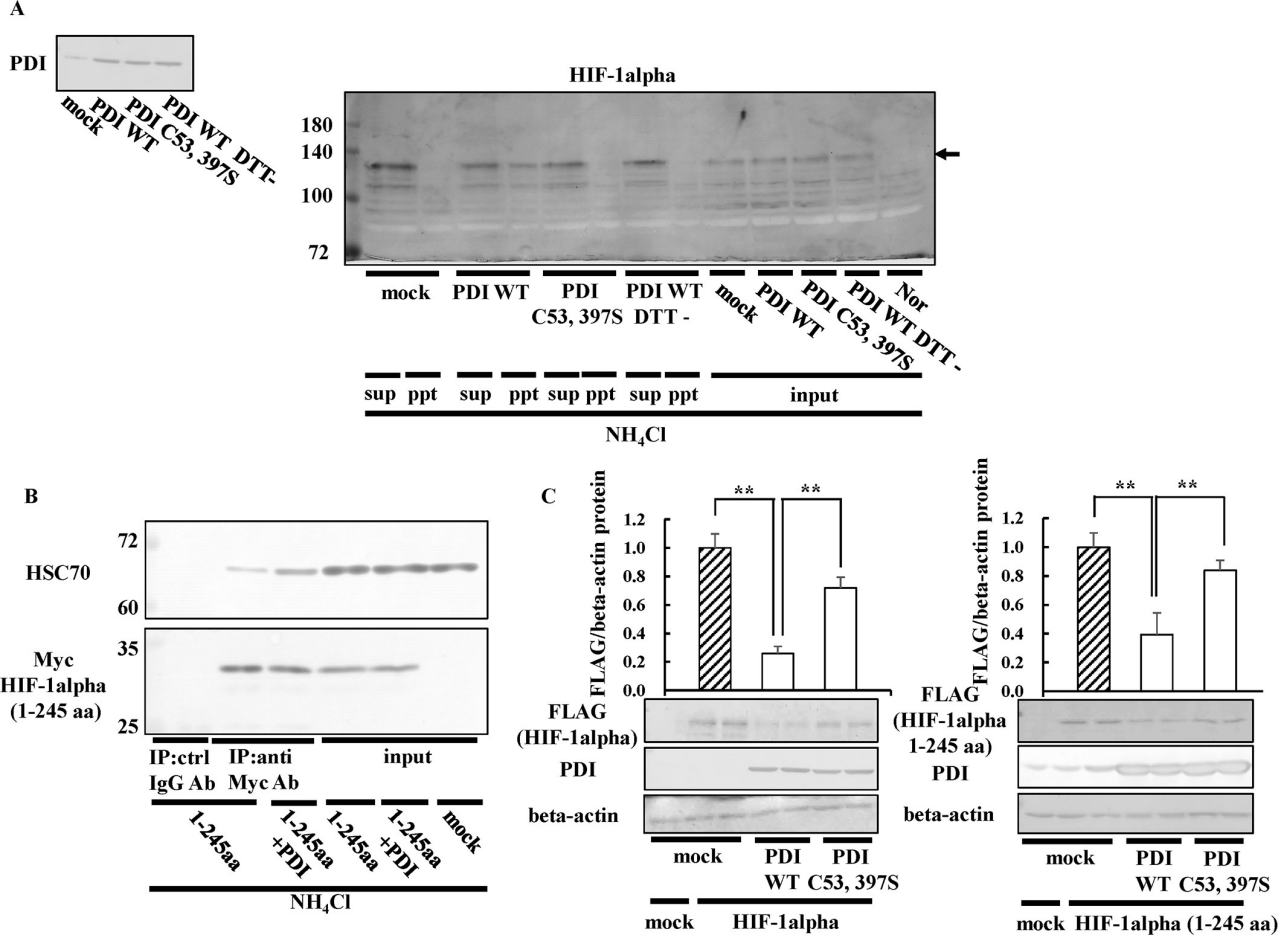
Effects of PDI on HIF-1alpha (1–245 aa) expression. (A) Hep3B cells overexpressing PDI WT or PDI C53, 397S were cultured under hypoxic conditions for 8 h in the presence of NH_4_Cl. The biotin-switch method was performed, and HIF-1alpha was detected by immunoblotting with the anti-HIF-1alpha antibody. (B) Hep3B cells overexpressing HIF-1alpha 1–245 aa or PDI were cultured for 8 h in the presence of NH_4_Cl. Cell extracts were subjected to immunoprecipitation using the anti-Myc antibody (1:1000 dilution). Precipitated proteins (15 or 3 μg) were separated by SDS-PAGE, and immunoblotting was performed with the anti-Myc or -HSC70 antibody. (C) HIF-1alpha full length or 1–245 aa/3×FLAG-pcDNA4 vector, or PDI WT, or PDI C53, 397S / pcDNA 3.1 (+) was transfected into Hep3B cells, and immunoblotting was performed with the anti-FLAG, -PDI, or-beta-actin antibody. The HIF-1alpha protein levels of mock cells under normoxic conditions were set to 1.0. Values are expressed as the mean ± S.D. (*error bars*) of three different plates. ctrl, control.  ** *p*<0.01, significantly different from mock cells.

**Fig 10:**
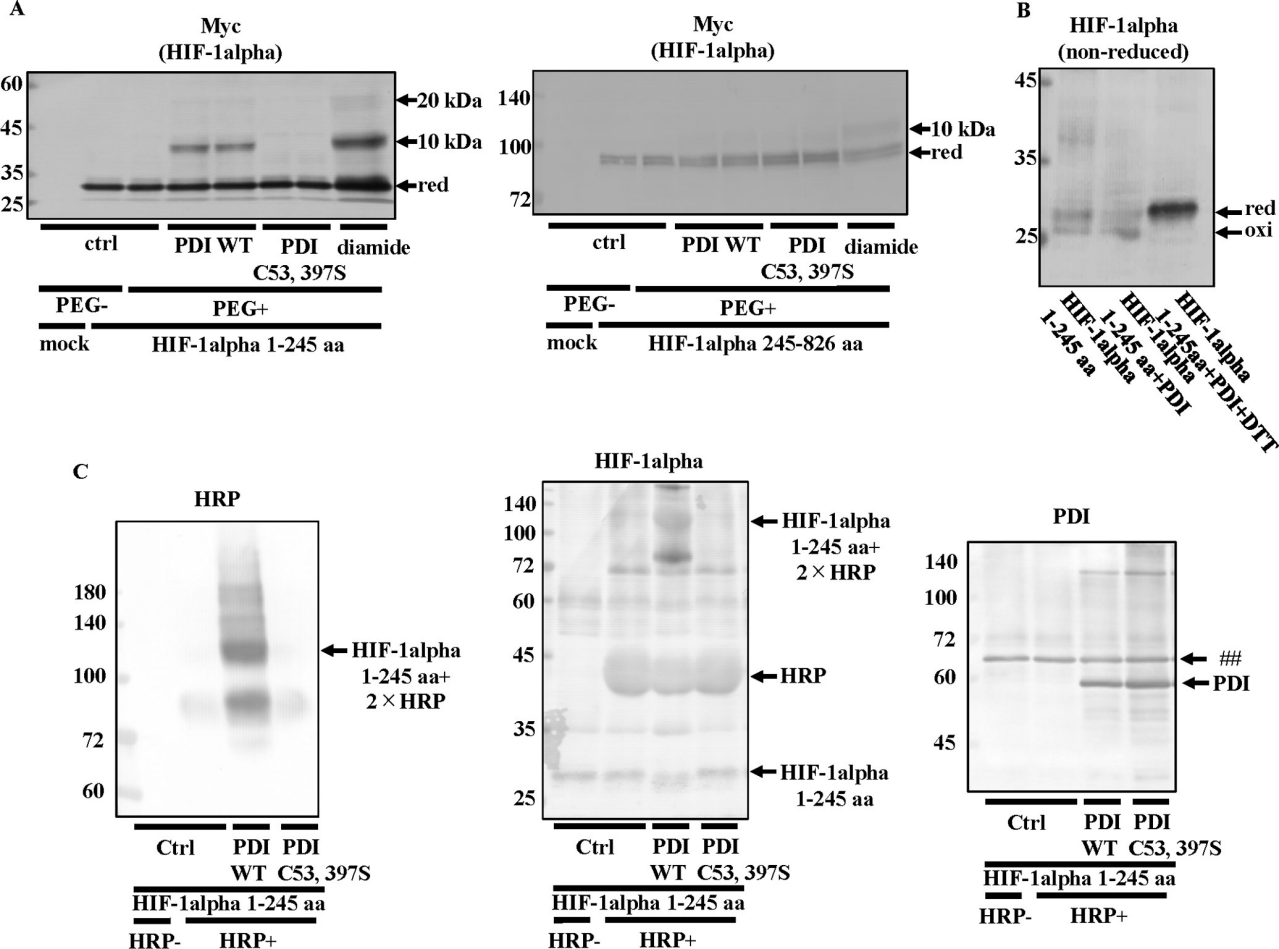
Detection of the HIF-1alpha (1–245 aa) redox state. (A) Hep3B cells overexpressing HIF-1alpha 1–245 aa or HIF-1alpha 245–826 aa were cultured under hypoxic conditions for 6 h, and 17.5 nM purified His-tagged PDI WT or PDI C53, 397S was added to the lysate from Hep3B cells. The redox states of proteins were detected using the same method as in Fig 4A. An up-shift in molecular weight by the binding of PEG-maleimide was detected by immunoblotting with the anti-Myc antibody. (B) 350 nM purified His-tagged PDI was added to 350 nM His-tagged HIF-1alpha 1–245 aa, and proteins were incubated in the presence or absence of 10 mM DTT. Precipitated proteins were incubated with 50 mM NEM, and immunoblotting was performed with the anti-HIF-1alpha antibody. (C) 350 nM purified His-tagged PDI WT or PDI C53, 397S was added to 350 nM His-tagged HIF-1alpha 1–245 aa, and proteins were incubated with 20 mM NEM. After the removal of NEM, proteins were incubated with 1 mM DTT. Precipitated proteins were incubated with HRP Maleimide Conjugate. An up-shift in molecular weight by the binding of HRP was detected by immunoblotting with or without the anti-HIF-1alpha antibody. ##, non-specific band.

In further experiments on the oxidation of HIF-1alpha by PDI, we examined the HIF-1alpha redox state using the biotin-switch assay (Fig 9A). We detected the HIF-1alpha band in the pull-down fraction of PDI-overexpressing cells, but not PDI C53, 397S-overexpressing cells in the presence of NH_4_Cl (Fig 9A). Since we did not observe the HIF-1alpha band in the pull-down fraction of non-reducing PDI-overexpressing cells, we confirmed that biotin-Sepharose bound to cysteine residues synthesized by reduction (Fig 9A). Hubbi *et al*. suggested that the amino acid residues 1–329 of HIF-1alpha (1–329 aa) interact with HSC70 [53]. Therefore, we investigated the effects of PDI on the interaction between HIF-1alpha 1–245 aa and HSC70. The results obtained showed that HSC70 also interacted with HIF-1alpha 1–245 aa, and this interaction was enhanced in PDI-overexpressing cells (Fig 9B). We then examined whether HIF-1alpha 1–245 aa was degraded by the overexpression of PDI. Full-length HIF-1alpha containing the FLAG tag was degraded in PDI-overexpressing cells (Fig 9C). HIF-1alpha 1–245 aa and full-length HIF-1alpha were degraded by PDI (Fig 9C). These results suggested that HIF-1alpha 1–245 aa was sufficient to be recognized and degraded by HSC70. We then assessed the effects of PDI on the HIF-1alpha 1–245 aa and HIF-1alpha 245–826 aa redox states. An up-shifted band of HIF-1alpha 1–245 aa by 10 kDa was induced by the addition of purified His-tagged PDI WT, but not PDI C53, 397S (Fig 10A). On the other hand, an up-shifted band of HIF-1alpha 245–826 aa by 10 kDa following the addition of purified His-tagged PDI WT was only slightly detected (Fig 10A). These results suggested that PDI directly oxidized HIF-1alpha 1–245 aa and that HIF-1alpha 1–245 aa was more important than HIF-1alpha 245–826 aa in the regulation of HIF-1alpha by PDI. However, we cannot exclude the possibility that HIF-1alpha formed a dimer and that PEG-maleimide bound to other amino acid residues. Therefore, we examined the redox state of HIF-1alpha 1–245 aa using NEM *in vitro* (Fig 10B). Since NEM does not bind to disulfide bonds in HIF-1alpha 1–245 aa, oxidized HIF-1alpha 1–245 aa maintained a closed conformation and then down-shifted under non-reducing conditions. We added purified His-tagged PDI to purified His-tagged HIF-1alpha 1–245 aa (Fig 10B). The down-shifted band was increased by the addition of purified His-tagged PDI (Fig 10B). These results indicated that PDI directly formed intramolecular disulfide bonds in HIF-1alpha 1–245 aa. We found an up-shifted band of HIF-1alpha 1–245 aa following the addition of purified His-tagged PDI WT using PEG-maleimide. Therefore, we also examined the *in vitro* effects of PDI on the redox state of HIF-1alpha 1–245 aa using maleimide-activated HRP, which recognizes cysteine residues (Fig 10C). Up-shifted bands are detected without an antibody when HRP binds to cysteine residues. We detected an up-shifted band of purified His-tagged HIF-1alpha 1–245 aa following the addition of purified His-tagged PDI WT, but not PDI C53, 397S without an antibody (Fig 10C). These up-shifted bans were detected with an anti-HIF-1alpha antibody (Fig 10C), suggesting that these bands were up-shifted HIF-1alpha 1–245 aa. We added equal amount of purified His-tagged PDI WT and PDI C53, 397S (350 nM) to His-tagged HIF-1alpha 1–245 aa (Fig 10C). These results also indicated that PDI induced disulfide bonds in HIF-1alpha 1–245 aa.

### Identification of critical cysteine residue in HIF-1alpha regulation by PDI

**Fig 11:**
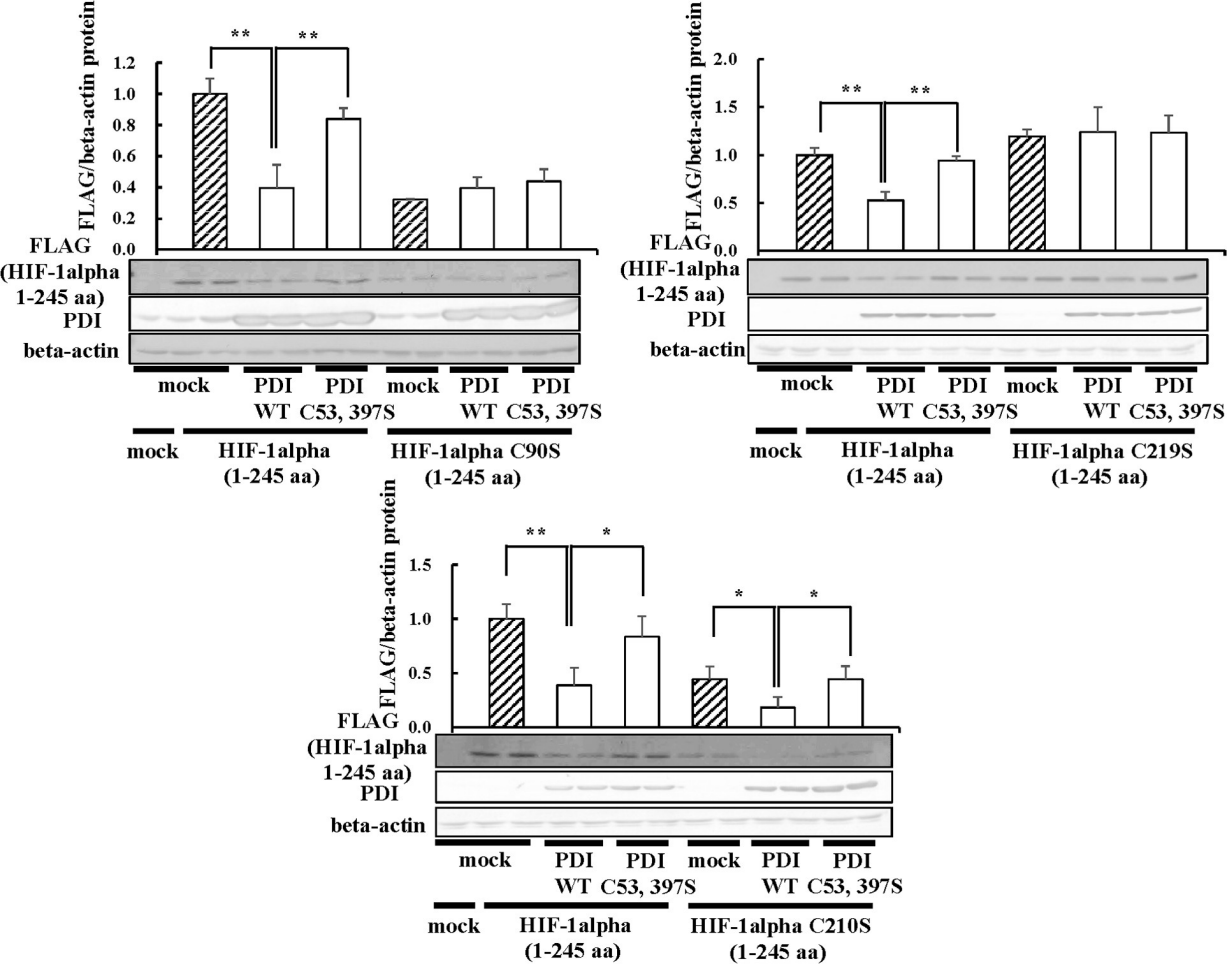
Identification of critical cysteine residue in HIF-1alpha regulation by PDI. HIF-1alpha C90S, HIF-1alpha C210S, or HIF-1alpha C219S/3×FLAG-pcDNA4 vector, or PDI WT, or PDI C53, 397S / pcDNA 3.1 (+) vector was transfected into Hep3B cells, and immunoblotting was performed with the anti-FLAG, -PDI, or-beta-actin antibody. The HIF-1alpha protein levels of mock cells under normoxic conditions were set to 1.0. Values are expressed as the mean ± S.D. (*error bars*) of three different plates. ctrl, control.  * *p*<0.05; ** *p*<0.01, significantly different from mock cells.

Next, we investigated important cysteine residues in the formation of intramolecular disulfide bonds in HIF-1alpha 1–245 aa. Seven cysteine residues are present in HIF-1alpha 1–245 aa. We speculated that the distance between two cysteine residues is important for the formation of disulfide bonds. Cys-90 is far from other six cysteine residues. Cys-210 and Cys-219 are close to Cys-194 and Cys-224, respectively. Therefore, we focused on three cysteine residues, Cys-90, Cys-210, and Cys-219. We prepared three mutants of HIF-1alpha 1–245 aa, in which the 90th, 210th, or 219th cysteine residue was replaced by serine residue, respectively, and these three mutants were overexpressed in Hep3B cells. The results showed that the overexpression of PDI did not affect the expression of HIF-1alpha C90S and HIF-1alpha C219S but not HIF-1alpha C210S (Fig 11). These results indicated that Cys-90 and Cys-219 in HIF-1alpha 1–245 aa might be important for formation of disulfide bonds by PDI.

## Discussion

The proliferation rate of cancer cells is higher than that of normal cells; therefore, increases in PDI expression levels are required for their growth. Therefore, PDI is regarded as a promising target for cancer therapy; however, the underlying molecular mechanisms remain unclear. Since HIF-1alpha is a critical factor in cancer progression, we elucidated the relation between PDI and HIF-1alpha in cancer cells. As a results, PDI decreased HIF-1alpha expression levels and transcriptional activity. In the present study, we investigated the mechanisms underlying the down-regulation of HIF-1alpha by PDI.

We previously reported that the overexpression of PDI suppresses the transcriptional activity of TR in GH3 cells [36]. The oxidation of Ref-1 by PDI inactivates Ref-1, resulting in the suppression of TR transcriptional activity [36]. Ref-1 has been shown to regulate several transcription factors, including HIF-1alpha, NF-kappa B, and AP-1, by the reduction of these factors [37, 56]. The knockdown of Ref-1 did not affect the decreases observed in HIF-1alpha expression levels by PDI. We confirmed the direct interaction of endogenous PDI with HIF-1alpha by immunoprecipitation. These results suggested that PDI directly changed the redox state of HIF-1alpha. The 10-kDa up-shifted band of endogenous HIF-1alpha was detected following the overexpression of PDI, but not PDI C53, 397S, using 5-kDa PEG-maleimide, suggesting that a disulfide bond was formed by PDI. These results indicated that the formation of disulfide bonds by dithiol oxidation via PDI is important for the degradation of HIF-1alpha. The oxidized form of PDI is increased via an endogenous oxidation pathway. Inaba *et al*. showed that Ero-1, an oxidation enzyme, directly interacts with PDI and selectively oxidized it [50]. Ero-1 exhibits strong oxidation activity, even under severe hypoxic conditions, which is an oxygen concentration <1% [49]. The overexpression of Ero-1 increased the oxidized form of endogenous PDI under hypoxic conditions. Moreover, the overexpression of the active form of Ero-1, which maintains the oxidation activity of PDI, decreased HIF-1alpha expression levels more than Ero-1 WT, whereas the inactive form of Ero-1 did not. These results indicated that HIF-1alpha expression levels were regulated via the oxidation activity of PDI.

HIF-1alpha is mainly degraded via the proteasome pathway [1]. In the present study, we found that PDI did not decrease HIF-1alpha expression levels in the presence of NH_4_Cl. Hubbi *et al*. recently reported that HIF-1alpha is delivered to the surface of lysosomes by HSC70 and interactes with LAMP2, resulting in the degradation of HIF-1alpha by lysosomal enzymes [53]. PDI did not decrease HIF-1alpha expression levels in HSC70-knockdown cells. Moreover, the overexpression of PDI induced the formation of disulfide bonds in HIF-1alpha in HSC70-knockdown cells, suggesting that oxidized HIF-1alpha evaded degradation by the knockdown of HSC70. These results indicated that the formation of disulfide bonds in HIF-1alpha by PDI was important for the degradation of HIF-1alpha via HSC70.

Although PDI has the KDEL sequence, an ER retention signal sequence, PDI localized in the cytosol, cell surface, and nucleus [33]. Na *et al*. suggested that cytosolic PDI is cleaved by caspase-3 and -7 and suppresses apoptotic cell death [57]. Wroblewski *et al*. suggested that cytosolic PDI in the liver cooperates with insulin-degrading enzyme and regulates metabolism of insulin [58]. These studies demonstrated that specific localization of PDI in cytosol plays a critical role in physiological processes. We also detected PDI in the cytosol fraction, and cytosolic PDI increased under hypoxic conditions. The mechanisms underlying the leakage of PDI into these fractions currently remain unclear; however, this result indicated that PDI affected HIF-1alpha expression levels in the cytosol. The levels of HIF-1alpha K719T, which was constitutively expressed in the cytosol, were reduced by PDI more strongly than HIF-1alpha WT. Therefore, the increase in cytosolic PDI may have been important for the degradation of HIF-1alpha.

In conclusion, we herein identified PDI as a novel negative regulator of HIF-1alpha in Hep3B cells. Previous studies demonstrated that the redox activity of PDI is required for the DNA binding of transcription factors, such as AP-1, NF-kappa B, and estrogen receptor alpha [34, 35]. The oxidation (formation of disulfide bonds) of transcription factors changes the conformation of their DNA-binding domain, resulting in the inhibition of DNA binding [59]. However, limited information is currently available on the effects of PDI on the expression of transcription factors. In the present study, we demonstrated that PDI directly oxidized HIF-1alpha and decreased its expression levels. The result showing that PDI regulated the expression of HIF-1alpha was novel. TRX increases the expression of HIF-1alpha, while the catalytic inactive mutant (TRX C32, 35S) decreases its expression; however, the effects of TRX on the redox state of HIF-1alpha remain unclear [29]. We investigated the effects of PDI on the HIF-1alpha redox state, and the results obtained showed that PDI oxidized HIF-1alpha. The oxidation of HIF-1alpha by PDI was important for the degradation of HIF-1alpha. To the best of our knowledge, this is the first study to detect dithiol-disulfide exchange in HIF-1alpha. The present results provide important insights into the mechanisms underlying the redox regulation of HIF-1alpha.

## Supporting information

S1 Raw images (PDF)
