# Supplementary material for: The regulation of Hypoxia-Inducible Factor-1 (HIF-1alpha) expression by Protein Disulfide Isomerase (PDI)
Source: PLoS One. 2021 Feb 4;16(2):e0246531. doi: 10.1371/journal.pone.0246531 (PMC7861413; doi:10.1371/journal.pone.0246531)

Raw images of Fig. 1

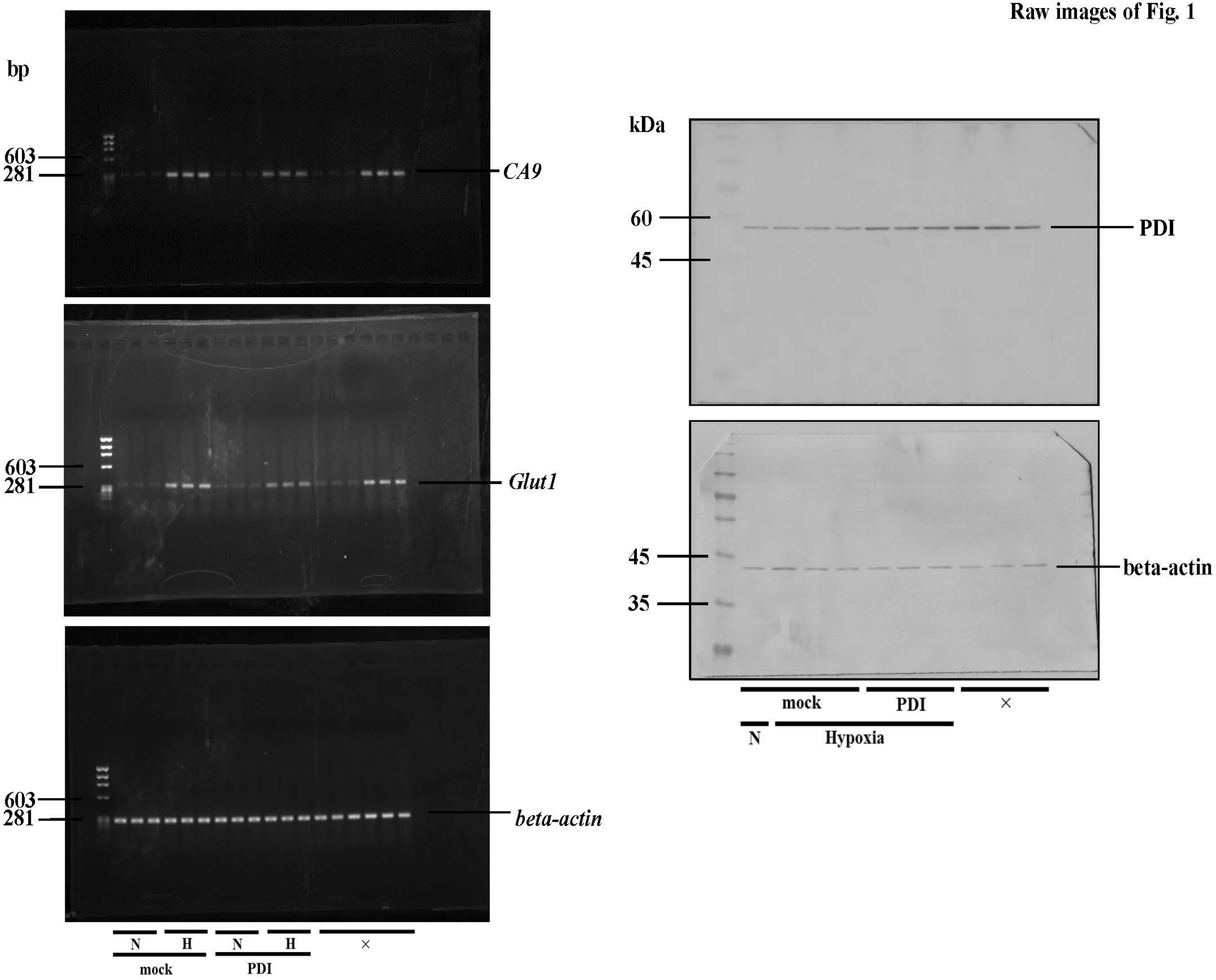

Raw images of Fig. 2

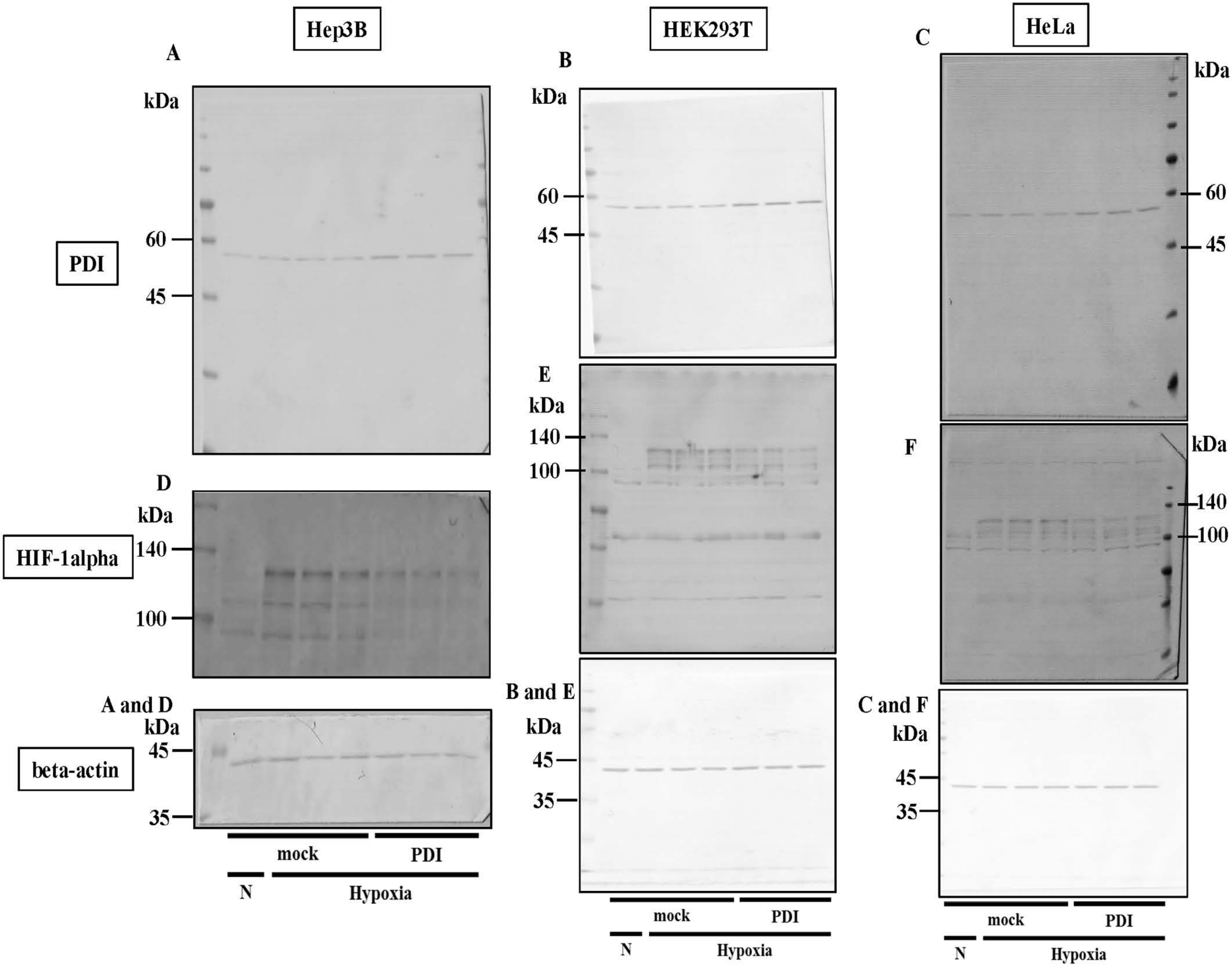

Raw images of Fig. 2

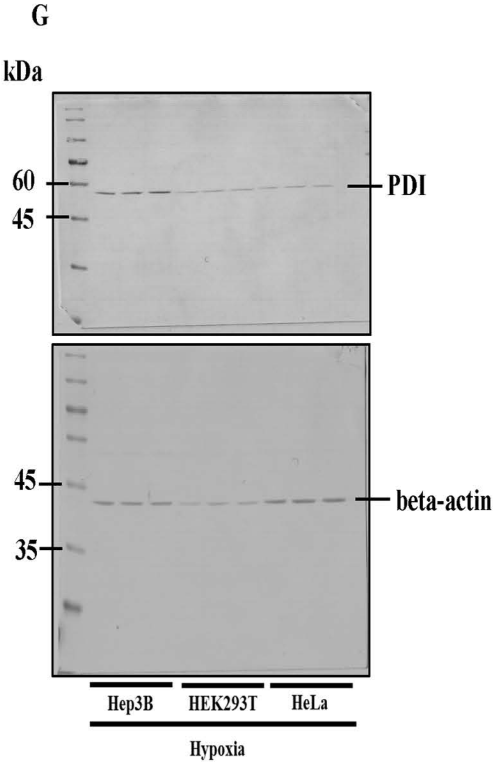

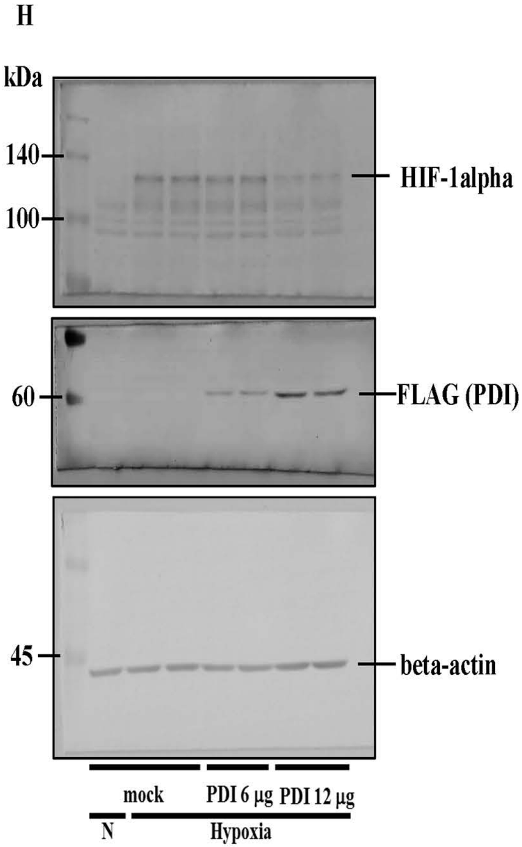

Raw images of Fig. 2

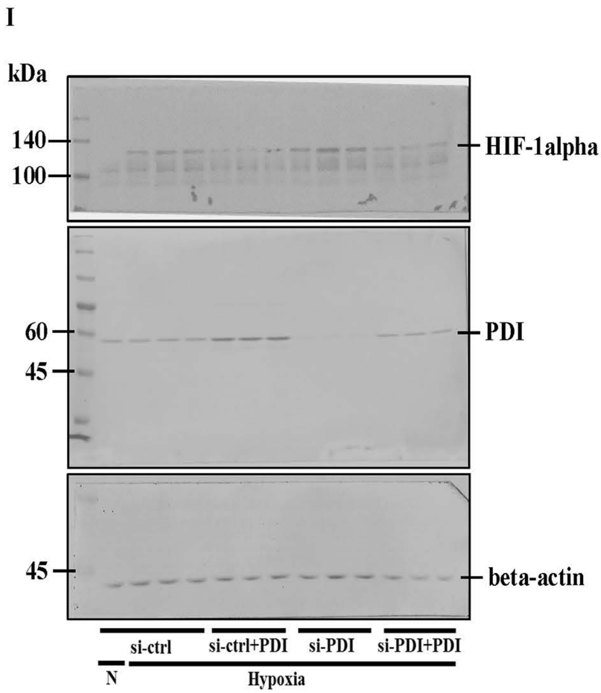

Raw images of Fig. 3

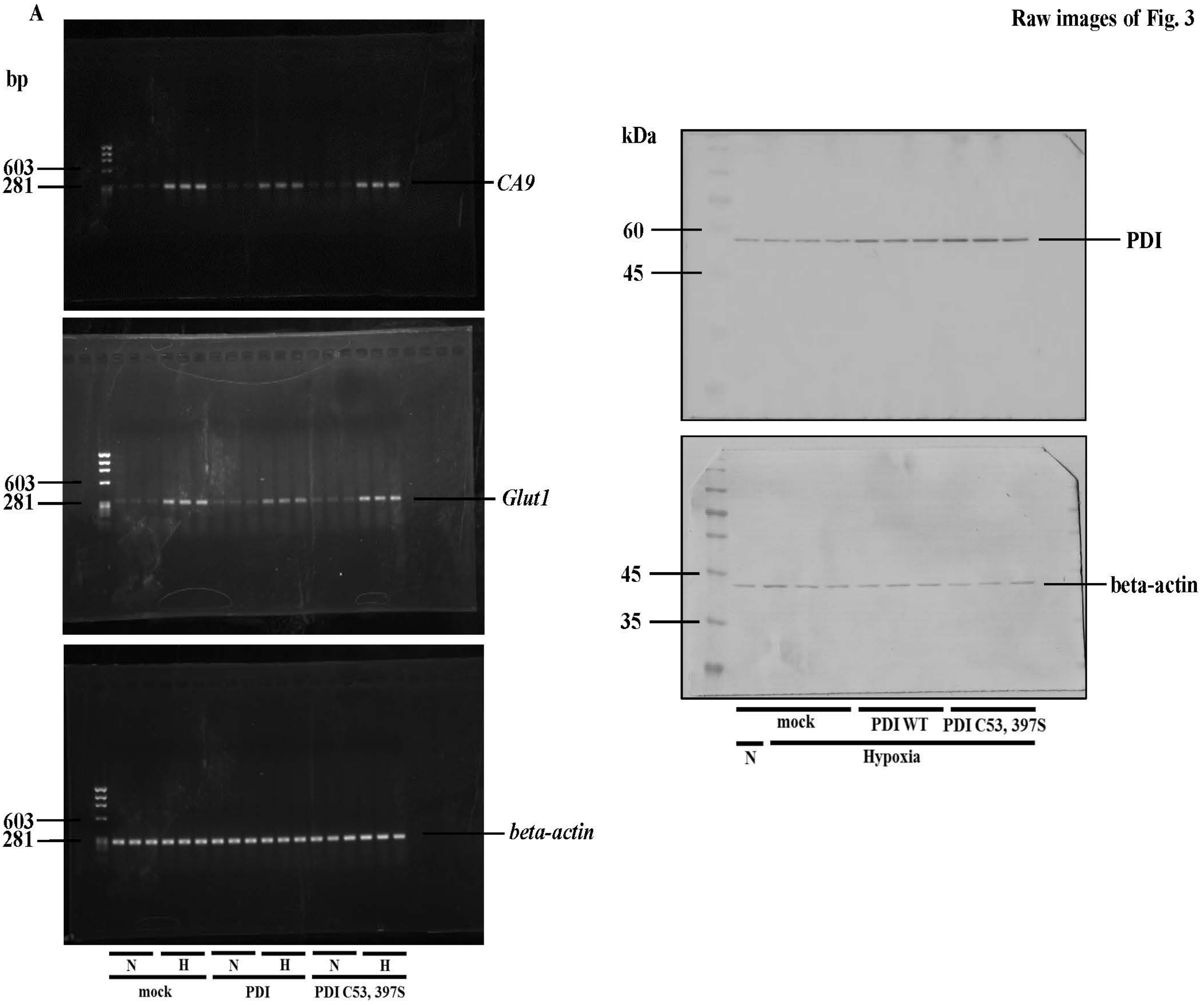

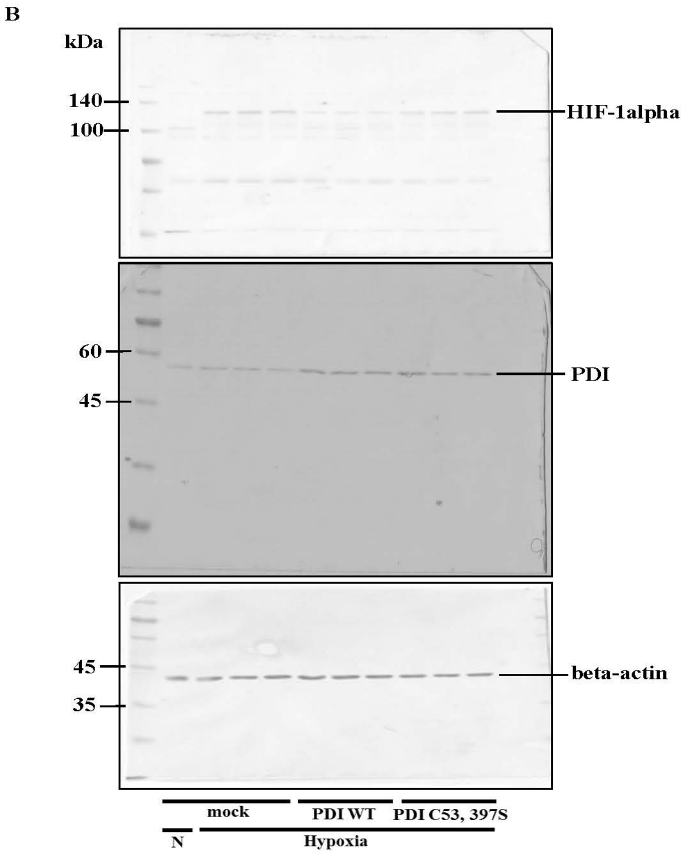
B
kDa
140
100
HIF-1alpha
60
PDI
45
45
beta-actin
35
mock
PDI WT
PDI C53, 397S
N
Hypoxia

Raw images of Fig. 4

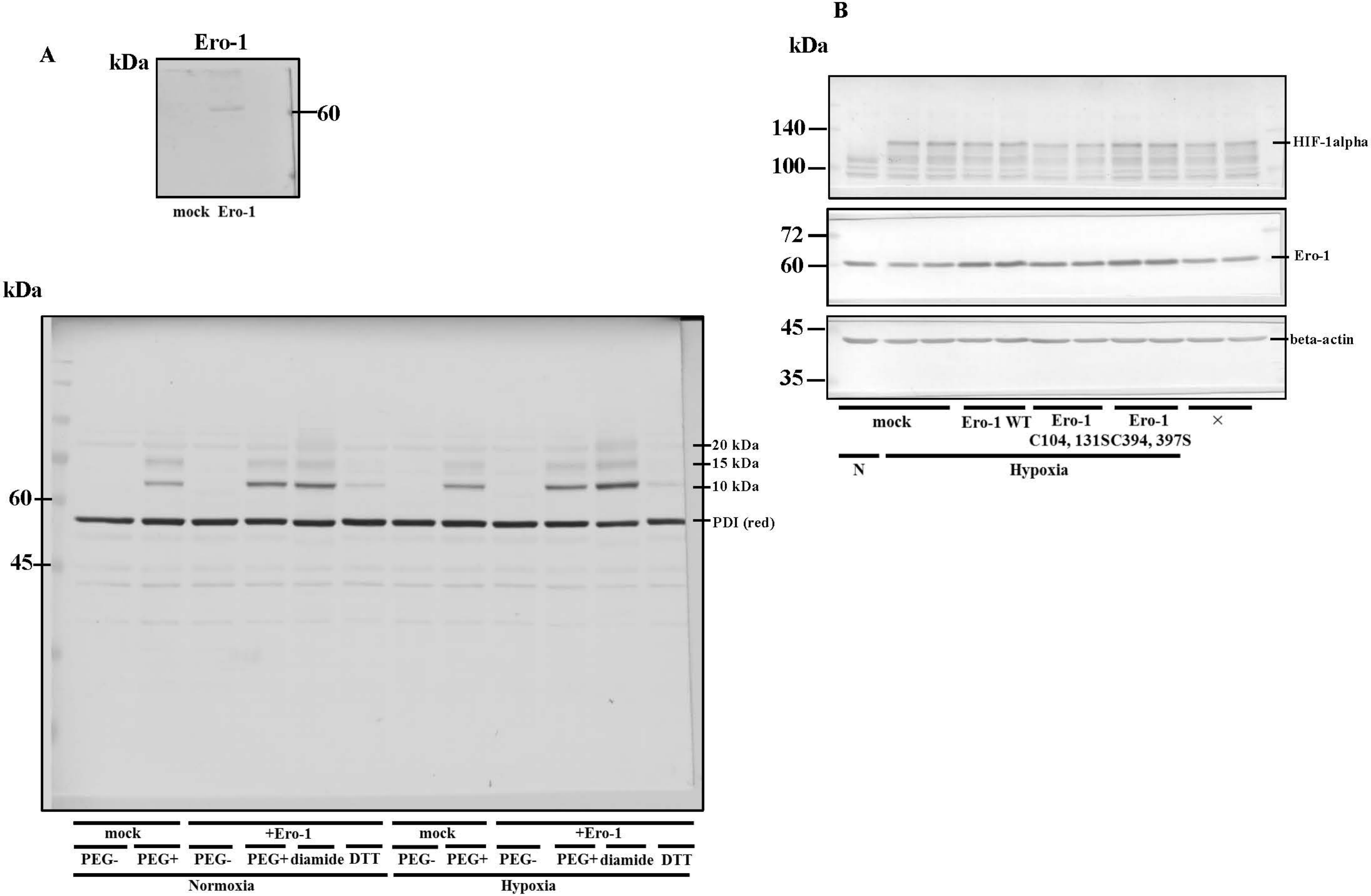

Raw images of Fig. 4

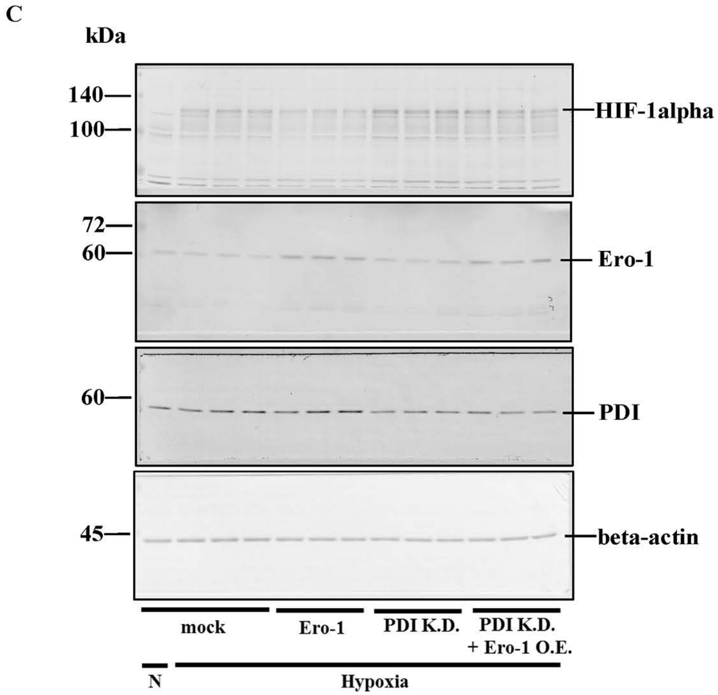

Raw images of Fig. 5

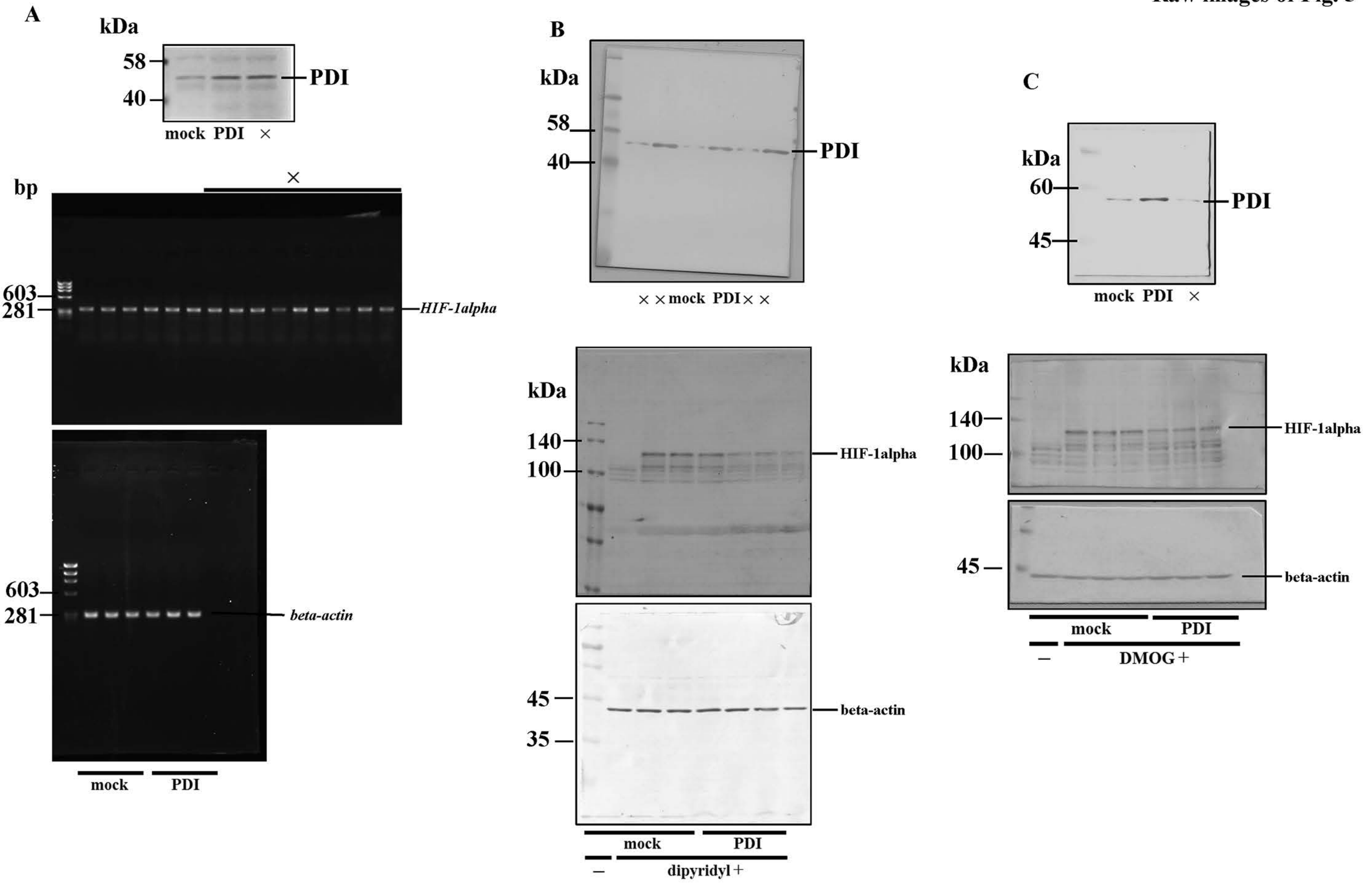

Raw images of Fig. 5

D

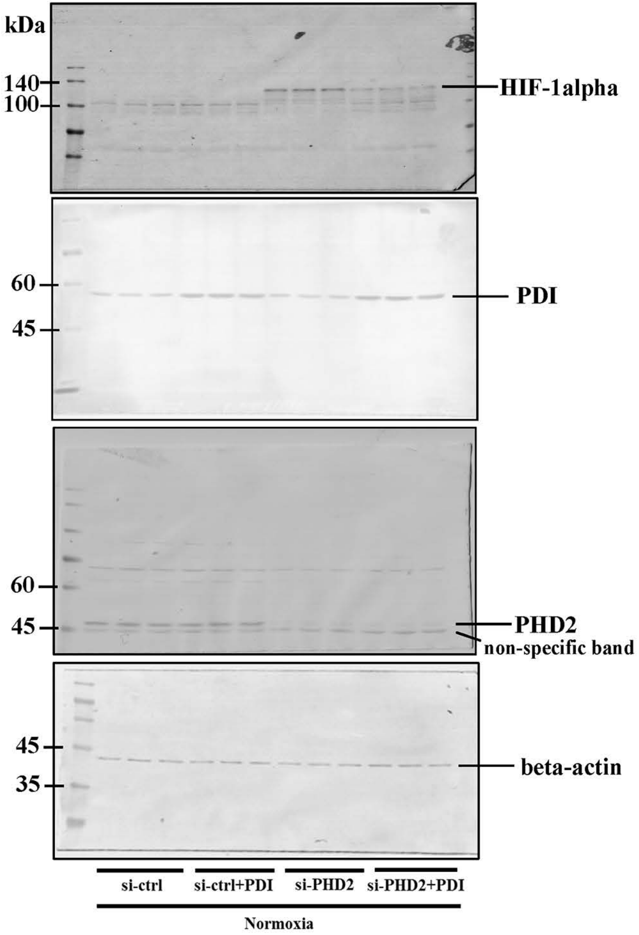

E

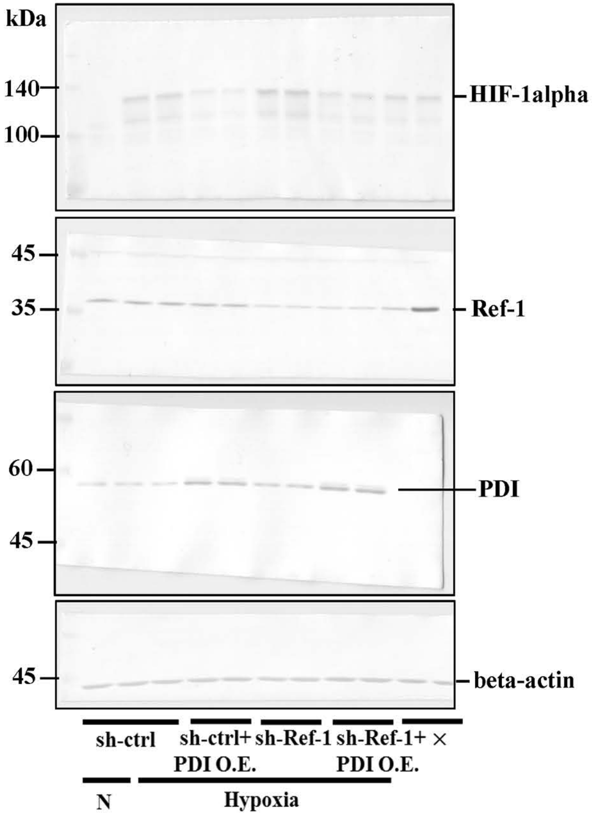

Raw images of Fig. 6

A

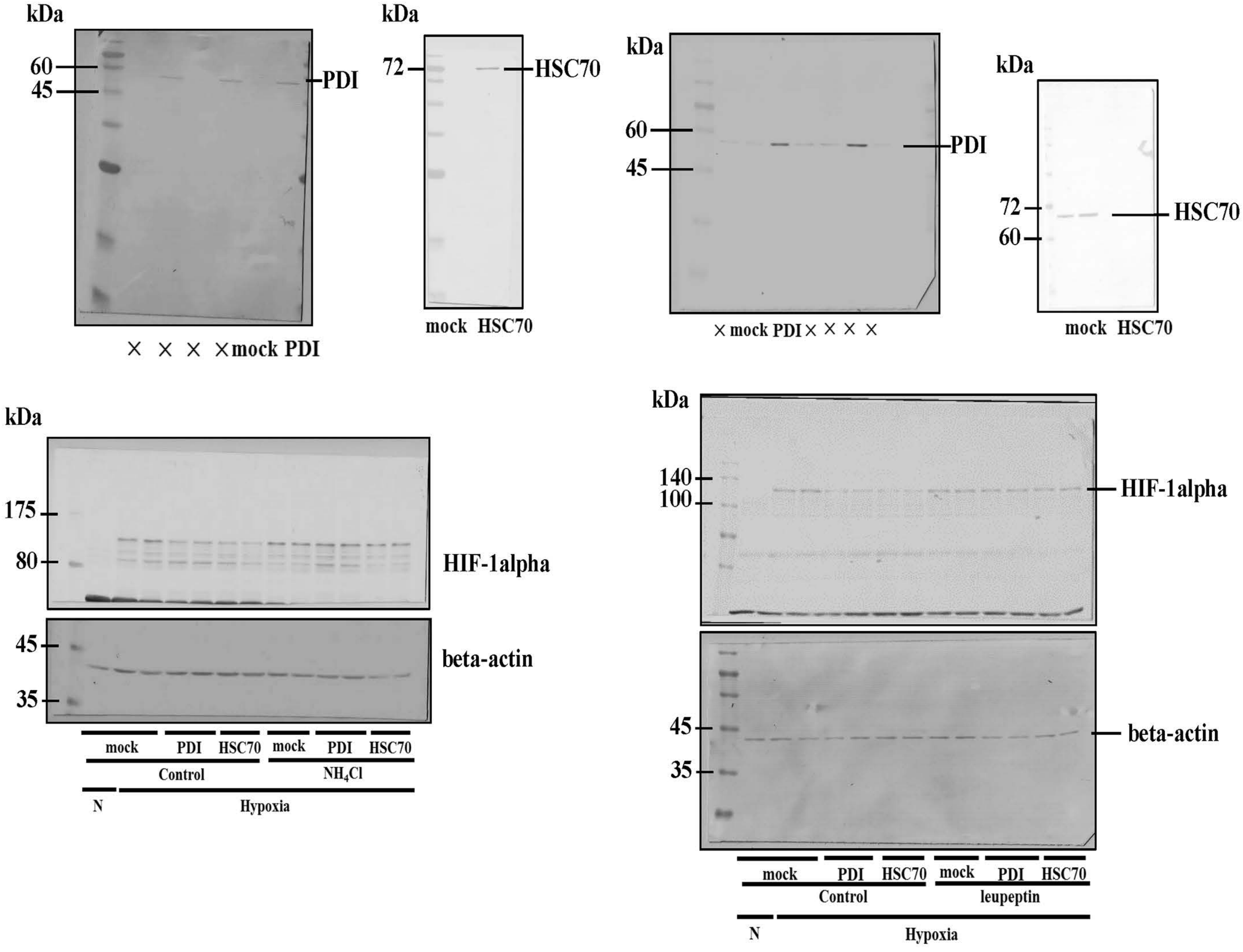

B

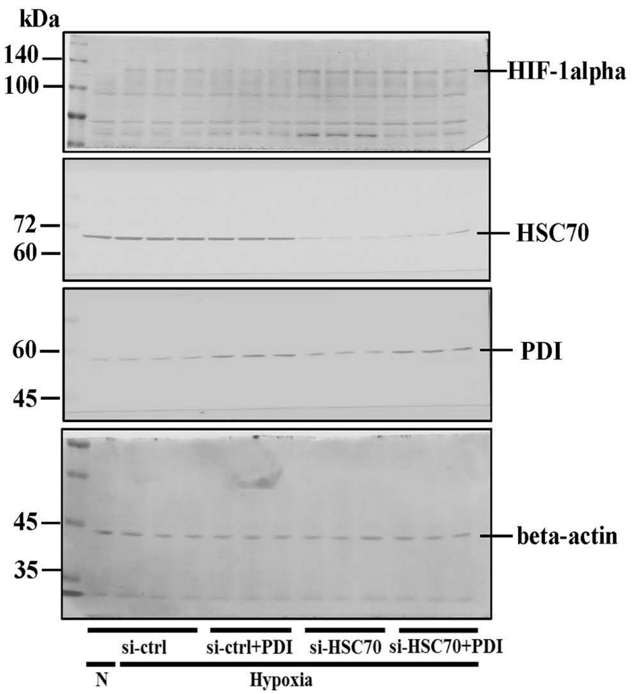
kDa
140
100
72
60
60
45
45
35
HIF-1alpha
HSC70
PDI
beta-actin
si-ctrl
si-ctrl+PDI
si-HSC70
si-HSC70+PDI
N
Hypoxia

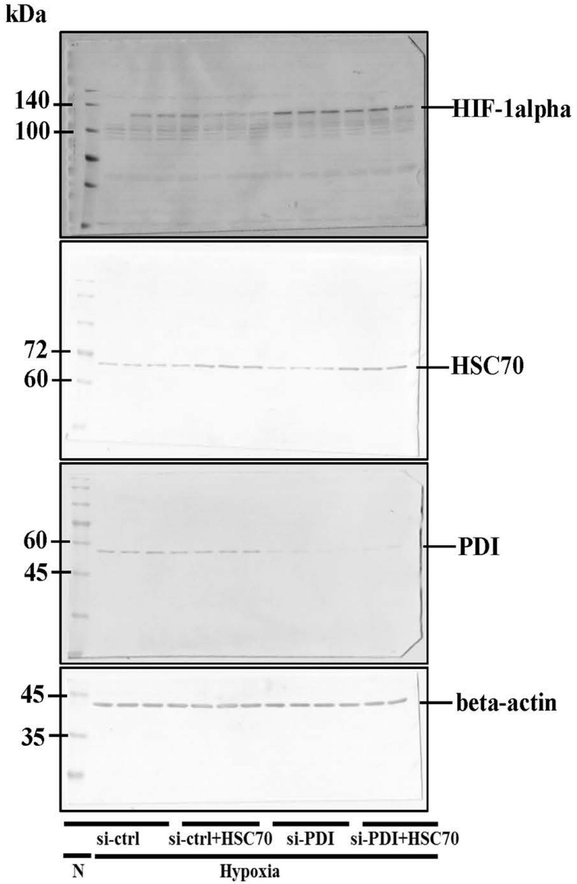
kDa
140
100
72
60
60
45
45
35
HIF-1alpha
HSC70
PDI
beta-actin
si-ctrl
si-ctrl+HSC70
si-PDI
si-PDI+HSC70
N
Hypoxia

Raw images of Fig. 6

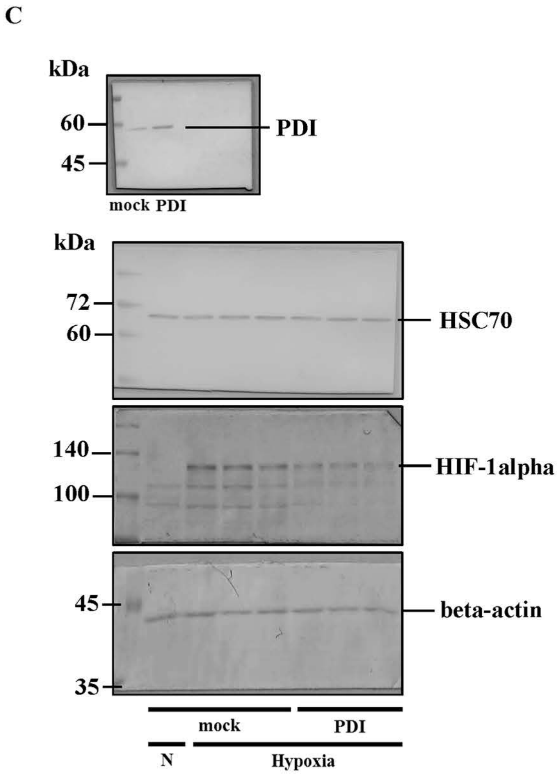

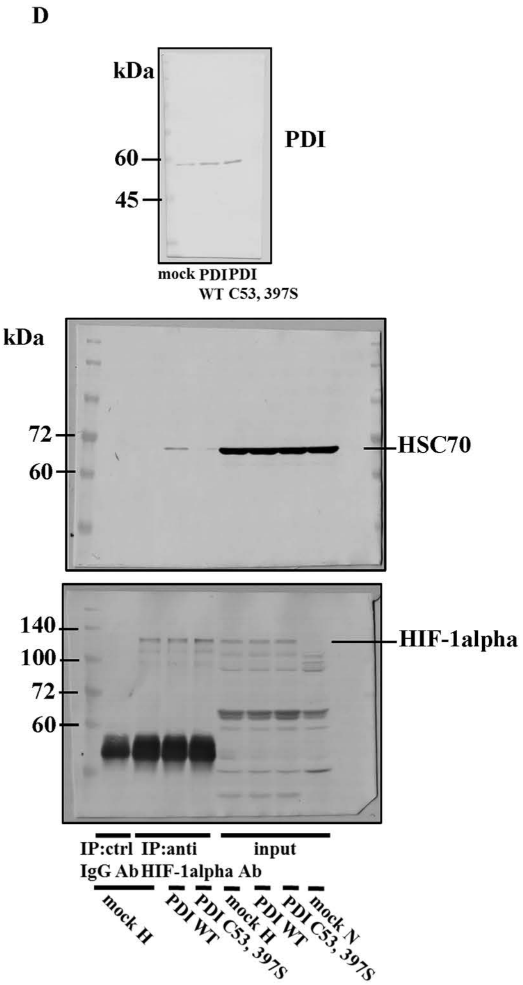

Raw images of Fig. 7

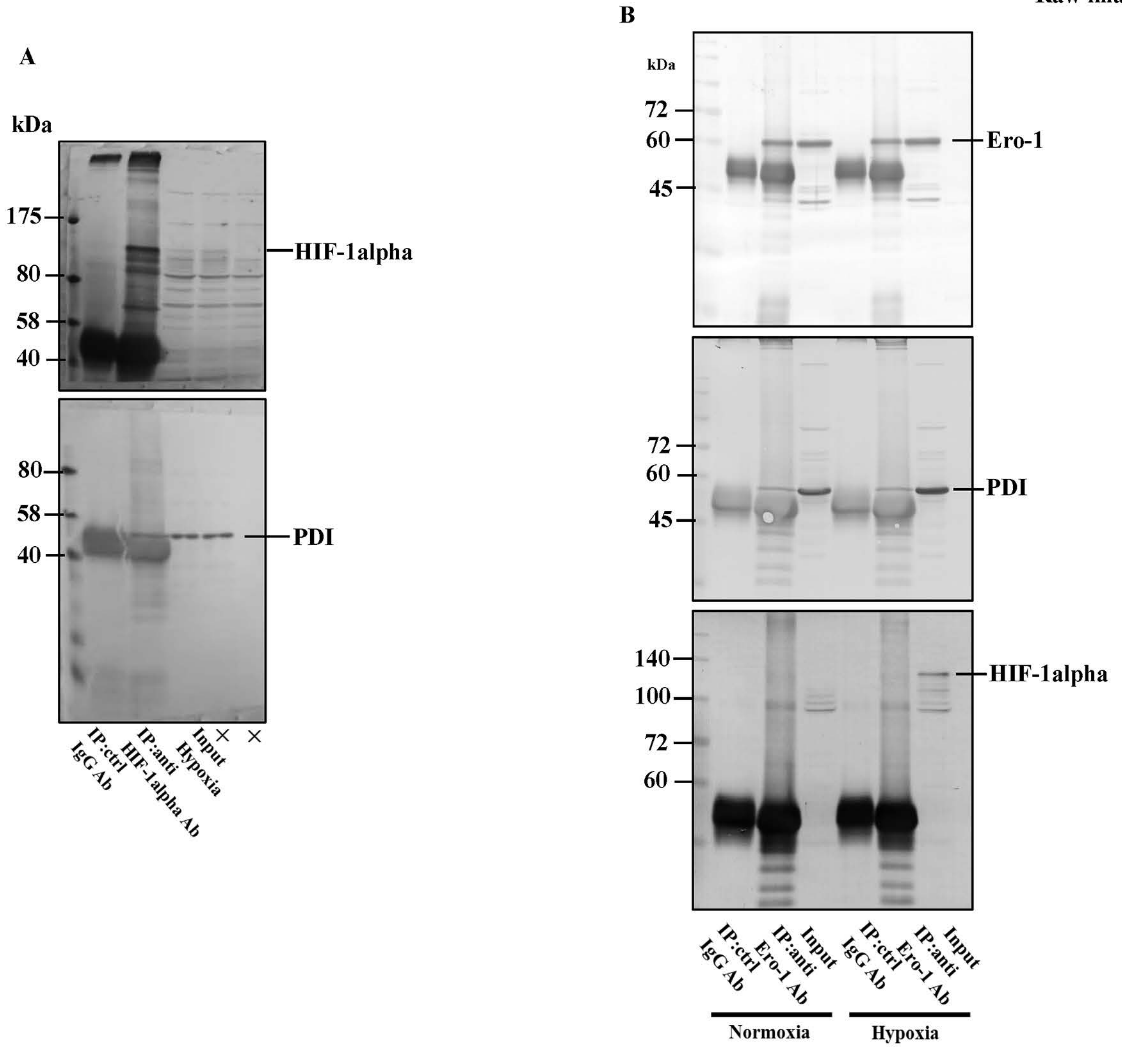

C

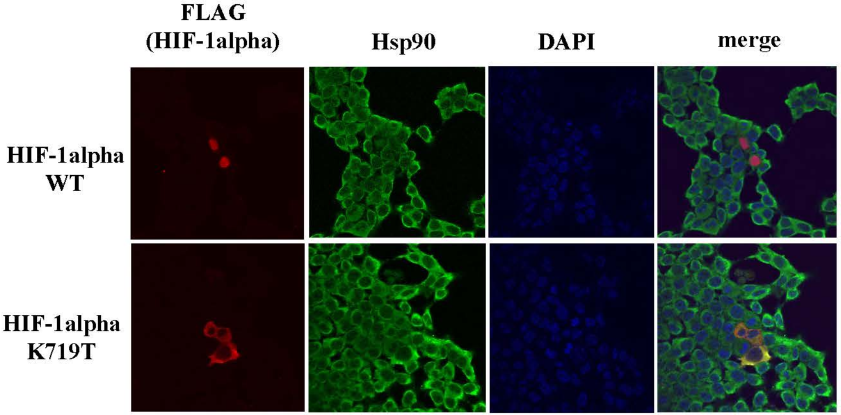
FLAG
(HIF-1alpha)
Hsp90
DAPI
merge
HIF-1alpha
WT
HIF-1alpha
K719T

D

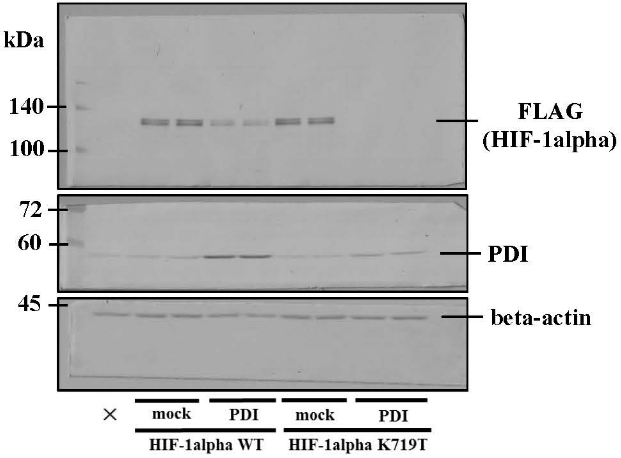
kDa
140
100
72
60
45
FLAG
(HIF-1alpha)
PDI
beta-actin
×
mock
PDI
mock
PDI
HIF-1alpha WT
HIF-1alpha K719T

E

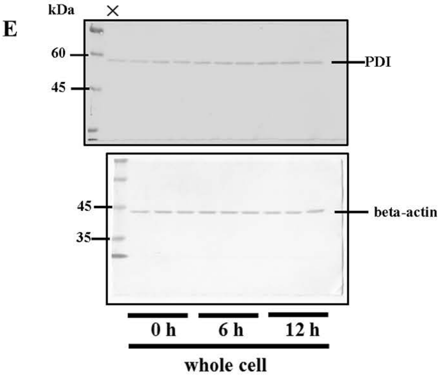
kDa
×
60
45
PDI
45
35
beta-actin
0 h
6 h
12 h
whole cell

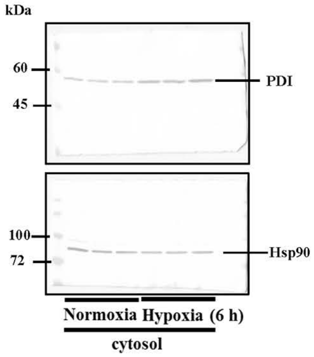
kDa
60
45
PDI
100
72
Hsp90
Normoxia
Hypoxia (6 h)
cytosol

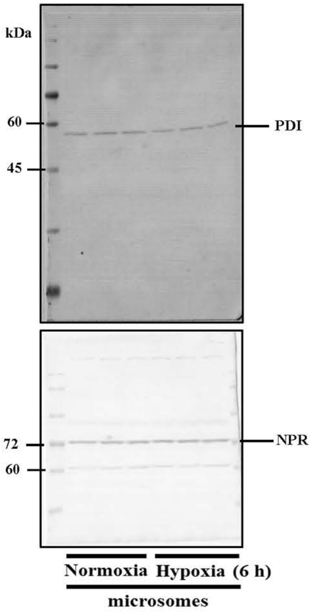
kDa
60
45
PDI
72
60
NPR
Normoxia
Hypoxia (6 h)
microsomes

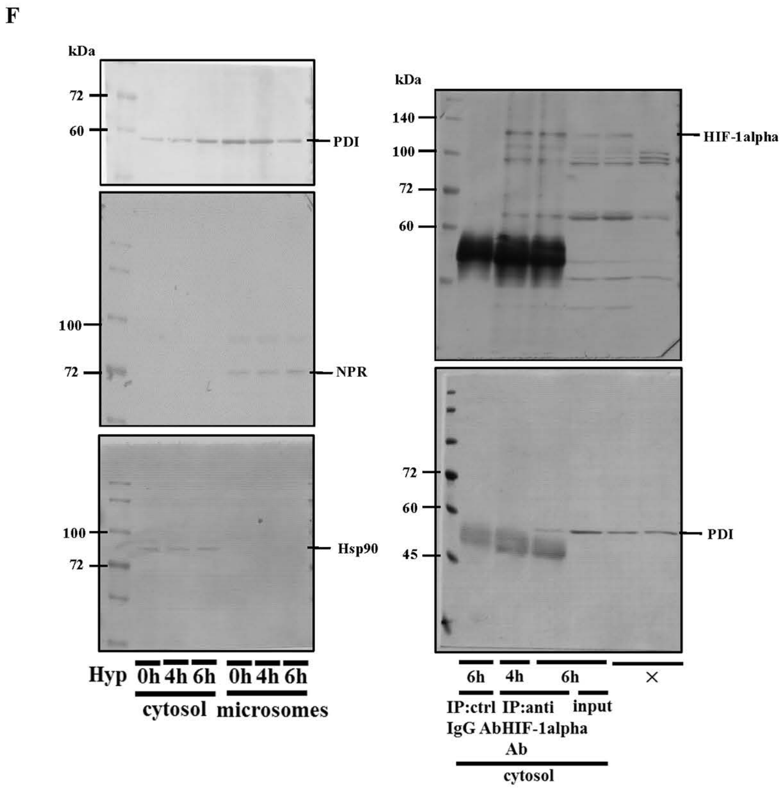
F
kDa
72
60
PDI
100
72
NPR
100
72
Hsp90
Hyp 0h 4h 6h 0h 4h 6h
cytosol microsomes
kDa
140
100
72
60
HIF-1alpha
72
60
45
PDI
6h 4h 6h ×
IP:ctrl IgG Ab
IP:anti HIF-1alpha Ab
input
cytosol

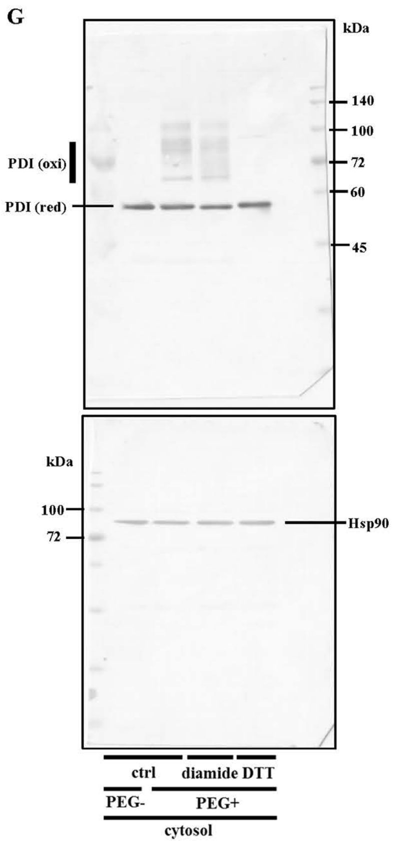
G
kDa
140
100
72
60
45
PDI (oxi)
PDI (red)
kDa
100
72
Hsp90
ctrl diamide DTT
PEG- PEG+
cytosol

H

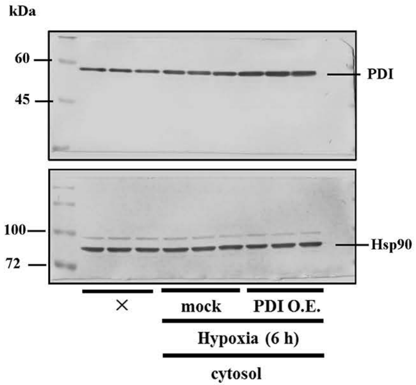

Fig. 8

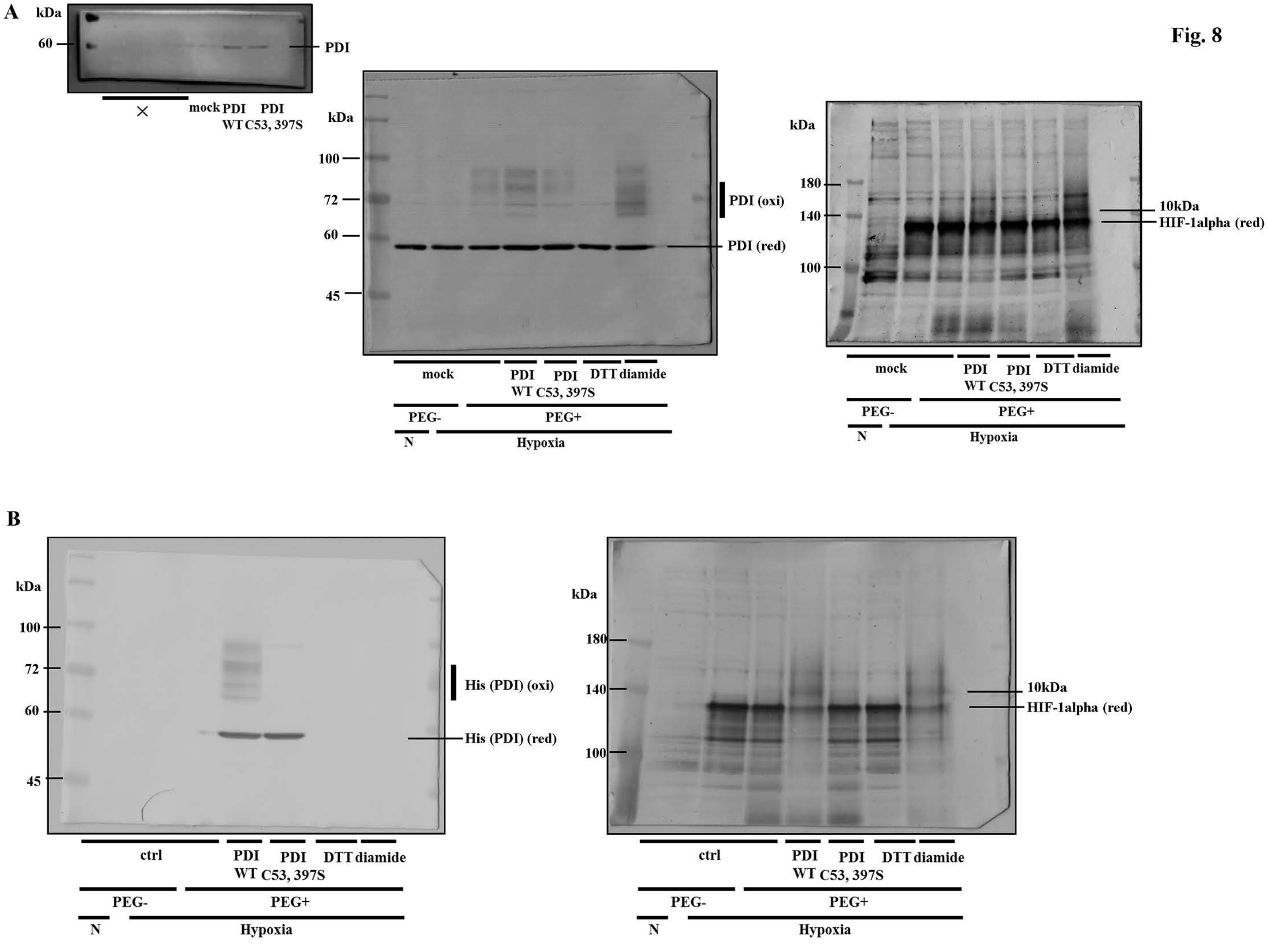
A
kDa
60
PDI
×
mock
PDI WT
PDI C53, 397S
kDa
100
72
60
45
PDI (oxi)
PDI (red)
mock
PDI WT
PDI C53, 397S
DTT
diamide
PEG-
PEG+
N
Hypoxia
kDa
180
140
100
10kDa
HIF-1alpha (red)
mock
PDI WT
PDI C53, 397S
DTT
diamide
PEG-
PEG+
N
Hypoxia
B
kDa
100
72
60
45
His (PDI) (oxi)
His (PDI) (red)
ctrl
PDI WT
PDI C53, 397S
DTT
diamide
PEG-
PEG+
N
Hypoxia
kDa
180
140
100
10kDa
HIF-1alpha (red)
ctrl
PDI WT
PDI C53, 397S
DTT
diamide
PEG-
PEG+
N
Hypoxia

C

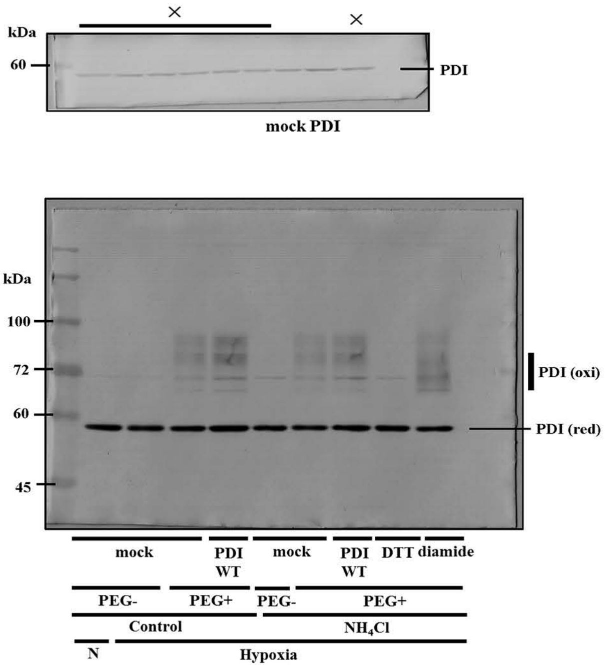

×
×
kDa
60
PDI
mock PDI
kDa
100
72
60
45
PDI (oxi)
PDI (red)
mock
PDI WT
mock
PDI WT
DTT
diamide
PEG-
PEG+
PEG-
PEG+
Control
NH_4Cl
N
Hypoxia

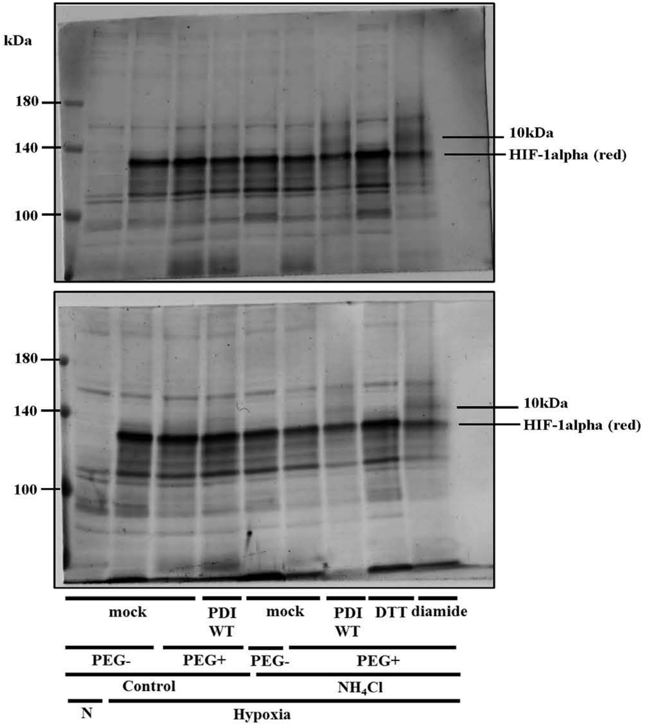

kDa
180
140
100
10kDa
HIF-1alpha (red)
180
140
100
10kDa
HIF-1alpha (red)
mock
PDI WT
mock
PDI WT
DTT
diamide
PEG-
PEG+
PEG-
PEG+
Control
NH_4Cl
N
Hypoxia

Raw images of Fig. 8

D

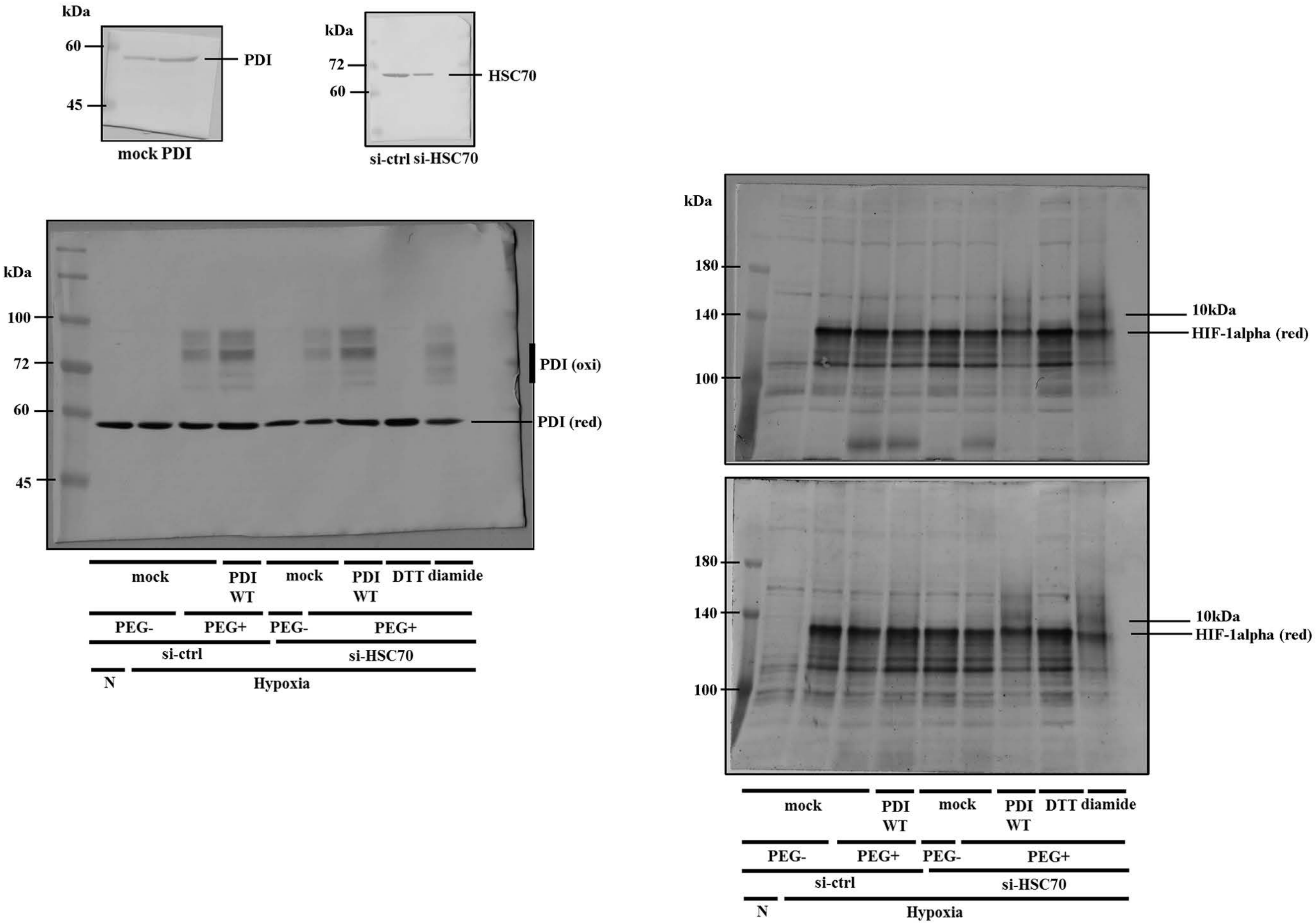

Raw images of Fig. 9

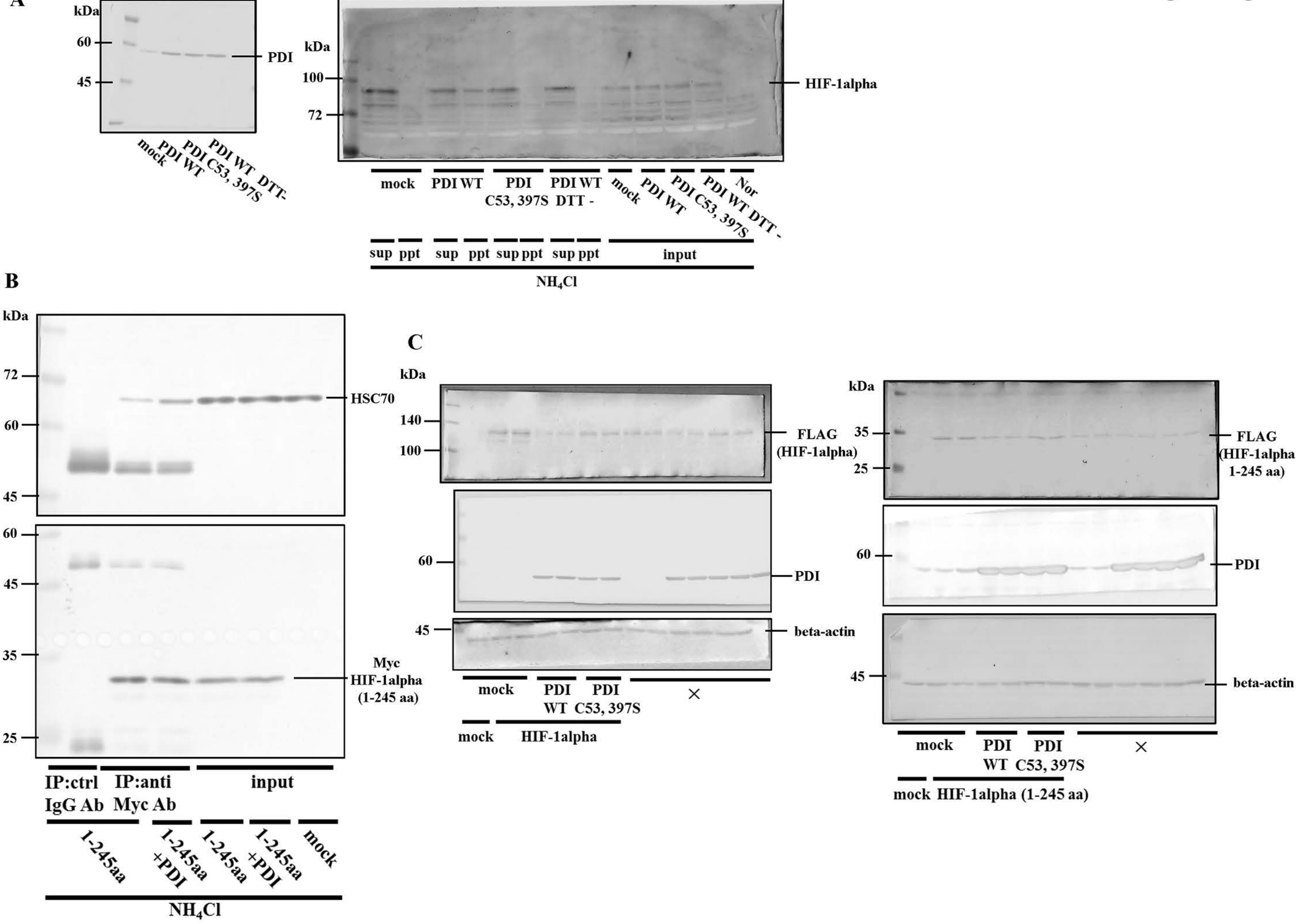

Raw images of Fig. 10

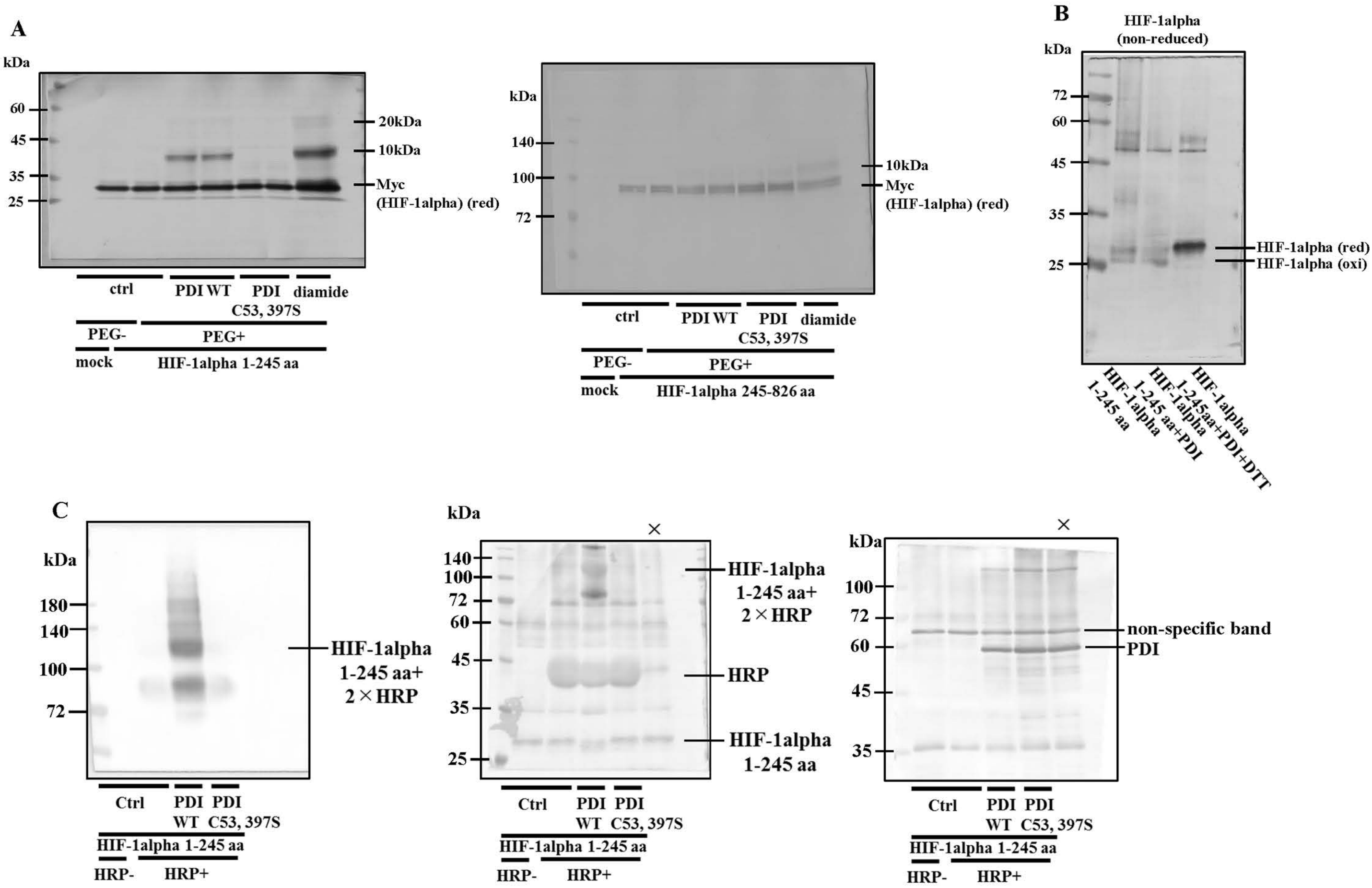

Raw images of Fig. 11

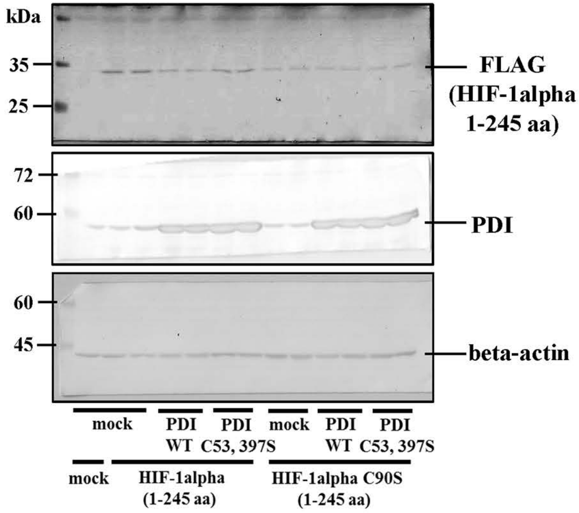

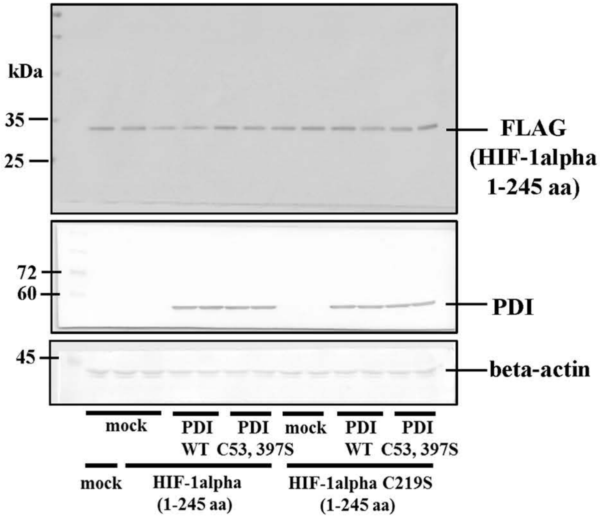

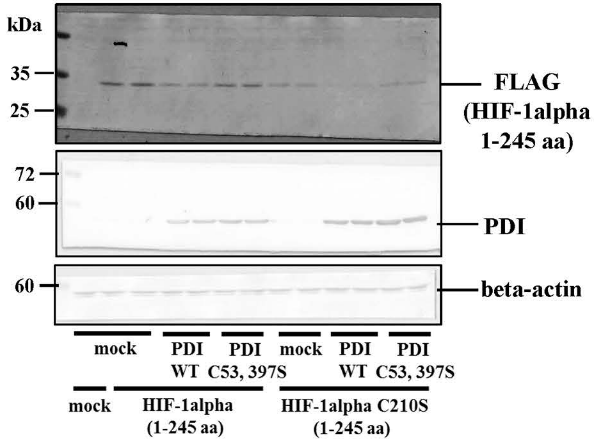

Supplement: S1 Raw images — (PDF) [file pone.0246531.s001.pdf]
